# Gut barrier-microbiota imbalances in early life lead to higher sensitivity to inflammation in a murine model of C-section delivery

**DOI:** 10.1186/s40168-023-01584-0

**Published:** 2023-07-03

**Authors:** M. Barone, Y. Ramayo-Caldas, J. Estellé, K. Tambosco, S. Chadi, F. Maillard, M. Gallopin, J. Planchais, F. Chain, C. Kropp, D. Rios-Covian, H. Sokol, P. Brigidi, P. Langella, R. Martín

**Affiliations:** 1 Microbiomics Unit, Department of Medical and Surgical Sciences, University of Bologna, 40138 Bologna, Italy; 2 INRAE, AgroParisTech, GABI, Paris-Saclay University, 78350 Jouy-en-Josas, France; 3 Animal Breeding and Genetics Program, Institute for Research and Technology in Food and Agriculture (IRTA), Torre Marimon, 08140 Caldes de Montbui, Spain; 4 INRAE, AgroParisTech, Micalis Institut,, Paris-Saclay University, 78350 Jouy-en-Josas, France; 5 CNRS, CEA, l’Institut de Biologie Intégrative de La Cellule (I2BC), Paris-Saclay University, 91405 Orsay, France; 6 Gastroenterology Department, Centre de Recherche Saint-Antoine, Centre de Recherche Saint-Antoine, CRSA, AP-HP, INSERM, Saint Antoine Hospital, Sorbonne Université, 75012 Paris, France; 7 Paris Centre for Microbiome Medicine (PaCeMM) FHU, Paris, France

**Keywords:** C-section delivery, Microbiota, Primary colonization, Early life, Inflammation, Gut barrier, Murine model

## Abstract

**Background:**

Most interactions between the host and its microbiota occur at the gut barrier, and primary colonizers are essential in the gut barrier maturation in the early life. The mother–offspring transmission of microorganisms is the most important factor influencing microbial colonization in mammals, and C-section delivery (CSD) is an important disruptive factor of this transfer. Recently, the deregulation of symbiotic host-microbe interactions in early life has been shown to alter the maturation of the immune system, predisposing the host to gut barrier dysfunction and inflammation. The main goal of this study is to decipher the role of the early-life gut microbiota-barrier alterations and its links with later-life risks of intestinal inflammation in a murine model of CSD.

**Results:**

The higher sensitivity to chemically induced inflammation in CSD mice is related to excessive exposure to a too diverse microbiota too early in life. This early microbial stimulus has short-term consequences on the host homeostasis. It switches the pup’s immune response to an inflammatory context and alters the epithelium structure and the mucus-producing cells, disrupting gut homeostasis. This presence of a too diverse microbiota in the very early life involves a disproportionate short-chain fatty acids ratio and an excessive antigen exposure across the vulnerable gut barrier in the first days of life, before the gut closure. Besides, as shown by microbiota transfer experiments, the microbiota is causal in the high sensitivity of CSD mice to chemical-induced colitis and in most of the phenotypical parameters found altered in early life. Finally, supplementation with lactobacilli, the main bacterial group impacted by CSD in mice, reverts the higher sensitivity to inflammation in ex-germ-free mice colonized by CSD pups’ microbiota.

**Conclusions:**

Early-life gut microbiota-host crosstalk alterations related to CSD could be the linchpin behind the phenotypic effects that lead to increased susceptibility to an induced inflammation later in life in mice.

Video Abstract

**Supplementary Information:**

The online version contains supplementary material available at 10.1186/s40168-023-01584-0.

## Background

The intestinal barrier is one of the important factors to have been implicated in homeostasis as well as in disease induction. It is a functional unit composed of a physical barrier formed by monolayer of epithelial cells (including Paneth cells and goblet cells (GC)) and the mucus layer, a chemical layer composed of several molecules such as immunoglobulin A (IgA) and antimicrobial peptides (AMP) and a immune layer composed of immune cells [1]. It constitutes the first line of defence as it separates self from non-self and protects against external insults. In humans, dysregulation of this barrier has led to impaired permeability and dysfunction, unchaining diseases such as irritable bowel syndrome (IBS), food allergies, type 1 diabetes and obesity [2–4]. In the early life, the developing gut barrier is established in conjunction with the gut microbiota [5]. Indeed, the gut microbiota is considered as a part of the gut barrier (microbiota layer) as it performs several roles related to the barrier function [6]. Furthermore, several bacteria, including commensals, have been found to directly or indirectly modulate intestinal barrier function [7–9]. The gut microbiota is a dynamic community shaped by multiple factors throughout an individual’s life [10]. The mother–offspring vertical transmission of bacteria is the most important factor influencing microbial colonization patterns in the neonate [10]. Symbiotic microbes begin to colonize the neonate during delivery [11]. As the infant passes through the birth canal, they will be exposed first to microbes in the vagina, on the maternal skin, and in faeces and then to microbes in the outside environment [12, 13]. As the newborn is experiencing maternal microbial imprinting, which plays an important role in gut microbiota development in early life, any disruption of this vertical microbiota transmission will alter primary colonization in the neonate. During the perinatal period, C-section delivery (CSD) has a tremendous disruptive influence in the context of full-term deliveries [14–16], even independently of antibiotic exposure [16, 17]. These modifications have a profound impact on the host by changing primary colonization patterns [18, 19]. In humans, the early community of colonizers in CSD newborns is similar to microbes living on the mother’s skin and in the operating room with depleted levels of *Bacteroidetes* [18, 20]. Indeed, this disrupted transmission of maternal microbiota has been related to the higher presence of antimicrobial-resistant opportunistic pathogens with the consequent risk for the newborn health [17, 21].

It has been suggested that there is a critical period during early development where disruptions of microbiota-host interactions could irreversibly harm the host priming process, thus hampering the establishment of a healthy internal balance, i.e. homeostasis [18, 22]. Such disruptions are a major factors behind developmental problems and predispose hosts to develop altered gut barrier function and inflammatory diseases [23, 24], including inflammatory bowel diseases (IBD) and obesity [25–27]. Meta-analyses have shown that infants born through CSDs are at higher risk of developing IBD (mainly Crohn’s disease (CD)) [27]. Since these children have an altered bacterial community, it has been hypothesized that these alterations lead to differences in mucosal immune tolerance and disease risk in later life [28, 29].

Here, we postulate that the disruption of primary colonization caused by CSD triggers a breakdown in homeostasis along the gut barrier that feeds a vicious circle in which intestinal and systemic inflammation and immunological alterations could result in a higher predisposition to suffer inflammatory diseases, such as IBD. We tested this hypothesis by analysing the microbiota-host crosstalk in a murine model of CSD in conventional and ex-germ-free animals with a special focus on gut barrier structure and permeability. Our results demonstrate the effects of CSD on gut barrier impairments and susceptibility to future inflammatory-induced insult and the major role of the microbiota on these phenomena in mice. Finally, we found that, in our murine model, it is possible to counterbalance the observed negative effects related to CSD by supplementing mice with lactobacilli, the most affected bacterial group by CSD in mice.

## Methods

### C-section delivery experimental model

Conventional mice were maintained under specific pathogen-free (SPF) conditions at 21 °C in the animal facilities of the French National Research Institute for Agriculture, Food and Environment (IERP, INRAE Jouy-en-Josas, France). Animal care and work protocols were approved by the local regional ethical committee (*COMETHEA*) according to the EU directive 2010/63/EU.

Pregnant RjOrl:SWISS mice were purchased to Janvier (Le Genest Saint Isle, France) and housed individually under SPF conditions. On gestational day 19, pregnant females experienced hysterectomy, and litters were fostered by mothers that had delivered vaginally within the previous 12 h (CSD group). Control mice were delivered vaginally (VD group). To avoid bias, pregnant mice that delivered vaginally interchanged their litters.

### Lactobacilli isolation and culture conditions

Fresh faecal content of VD pups at weaning was recovered and immediately introduced in an anaerobic chamber with controlled atmosphere (N2 = 90%, CO2 = 5% and H2 = 5%) to proceed to the bacterial isolation. Briefly, faecal samples were homogenized in saline buffer and serial dilutions performed in order to plate dilutions 10^−8^ and 10^−9^ on MRS media (Difco) supplemented with cysteine (0.5 mg/ml; Sigma). Twenty colonies were recovered, identified by 16 s RNA gene sequencing by standard protocols and stocked at − 80C with 40% of glycerol. Four of them, 3 *Lactobacillus murinus* and 1 *Lactobacillus taiwanensis*, were employed for the ex-germ-free (GF) experiments.

Lactobacilli were growth on MRS medium supplemented with cysteine at 37 °C under anaerobic conditions. To prepare the force-feeding solution, an overnight liquid culture of each of them were centrifugated, washed and resuspended together in PBS to achieve a final concentration of 5 × 10^9^ CFU/ml.

### Ex-germ-free mice experiments

Experiments with ex-germ-GF mice were conducted at the ANAXEM platform (INRAE, Jouy-en-Josas). In a first experiment, 45 axenic mice (C3H) were distributed in 3 different isolators. Each isolator was divided in different conditions: 15 axenic mice, 15 mice force-feed with faecal contents of a pool of hysterectomized mice at weaning and 15 mice force-feed with faecal contents of vaginal delivery mice at weaning. The force-feed process was repeated two consecutive days to maximize the colonization process. Weight were recorded each 3 days, and mice were euthanized at 3 different timepoints (2, 7 and 14 days) to monitor the effects of microbiota colonization in recipient mice.

In a second experiment, 26 C3H GF mice were distributed in 3 isolators: one with 9 mice force-feed with faecal contents of a pool of hysterectomized mice at weaning, a second one with 9 mice force-feed with faecal contents of a pool of hysterectomized mice at weaning and 10^9^ CFU of 4 lactobacilli strains (200 µl of the lactobacilli pool prepared as stated above) and a last one with 8 mice force-feed with faecal contents of vaginal-delivered mice. Mice weight was recorded at least twice per week, and mice were euthanized at 14-day post-colonization.

### Inflammation protocols on conventional and ex-GF mice

For conventional mice, litters were weaned at 3 weeks and randomly distributed in cages of 5 animals divided in C-section or vaginal groups. To induce acute inflammation, at 4 weeks of age (1 week after weaning), mice were anesthetized using an intraperitoneal (*i.p*) injection of 0.1% ketamine (Imalgene 1000, Merial, France) and 0.06% xylazine (Rompun, Bayer, France). Colitis was induced with an intrarectal dose of 200 mg/kg of 2,4-dinitrobenzene sulphonic acid (DNBS, Sigma-Aldrich, France) resuspended in 50 µl of 30% ethanol (EtOH) in PBS. To induce chronic colonic inflammation, 6-week old mice were submitted to a chronic administration of DNBS as previously described [30]. Briefly, mice were challenged with a first dose of DNBS (200 mg/kg). After 21 days of recovery, colitis was reactivated with a second dose of DNBS (200 mg/kg). For both colitis protocols, control mice received only 30% EtOH in PBS in the place of DNBS (vehicle groups). Weight loss was monitored for 3 days following the DNBS injections to assess possible clinical signs of distress. Due to the higher weight increase of CSD mice [16], in the case of acute colitis, weight prediction was performed according to Eisen and co-workers [31], and the weight lost was represented as the area under the curve (AUC) of the difference between expected and predicted weight. Colonic macroscopic and histological scores were determined as previously described [30].

For the experiment of colitis on ex-GF mice, 33 C3H mice were colonized under sterile conditions with the faecal contents of a pool of hysterectomized mice at weaning (supplemented or not with lactobacilli) or with faecal contents of vaginal delivery in ANAXEM facility as described above. Two-week post-colonization, mice were transferred to SPF conditions at IERP animal facilities where an acute colitis protocol was performed as explained above.

### Cytokines and total antioxidant capacity determinations

IL-1α, IL-1β, IL-6, IL-10, IL-12p70, IL-17A, IL-23, IL-27, MCP-1, IFN-β, IFN-γ, TNF-α and GM-CSF cytokines were determined in ileum, colon and serum samples with LEGENDplex™ mouse inflammation panel (BioLegend). Total antioxidant capacity was determined in serum samples by a colorimetric assay according to manufacturer instructions (Sigma-Aldrich).

### Quantification of myeloperoxidase (MPO) activity, lipocalin-2 and sCD14

Myeloperoxidase (MPO) activity was determined in colon and ileum samples as previously described [9]. Lipocalin-2 (Lcn-2) determination was performed on serum samples with the kit mouse lipocalin-2/NGAL DuoSet ELISA (R&D systems, Inc., USA & Canada) according to the manufacturer’s instructions. Plasma concentration of soluble CD14 (sCD14) was measured using ssCD14 ELISA kit (R&D).

### Histological analyses

Histological features were assessed using hematoxylin–eosin-saffron (HES) staining, Alcian blue (AB) staining and the periodic acid-Schiff (PAS) method in accordance with standard protocols [2, 32] in the Histology Facility of aBridge platform of UMR 1313 GABI. Images were analysed with Panoramic Viewer software.

### Lymphocyte populations in the spleen and mesenteric lymph nodes

After euthanasia, cell suspensions of spleen (at weaning and 5 days) and mesenteric lymphoid nods (only at weaning) were obtained by mechanically extrusion through a 40-μm nylon cell strainer (BD, Switzerland). Cells were washed through the strainer using 1 ml of Dulbecco’s Modified Eagle’s Medium (DMEM, Gibco, France) supplemented with 10% foetal bovine serum (FBS, Gibco). Erythrocytes were lysed by incubation with the red blood cell lysing buffer Hybri-Max (Sigma-Aldrich) according to manufacturer instructions. For each sample, aliquots of 10^6^ cells were transferred to two 96-well plates (Grenier, France). Following standard protocols as previously described [9, 30], cells were stained with one of the next: (i) anti-CD4-FITC, anti-CD3e-percp and anti-T-bet-PE; (ii) anti-CD4-FITC, anti-CD3e-percp and anti-Gata3-PE; (iii) anti-CD4-FITC, anti-CD3e-PerCP and anti-rorγ-PE; or (iv) anti-CD4-FITC, anti-CD3e-PerCP and anti-Foxp3-PE (all from eBioscience, France). All stainings were performed in the presence of CD16/CD32 (eBioscience). Samples were subsequently analysed using an Accuri C6 cytometer (BD). The data obtained from the cytofluorimetric analysis were processed using CFlowSampler software (BD).

### Short-chain fatty acids (SCFAs) determinations

In the day of the sacrifice, caecal samples were collected and put in liquid nitrogen at − 80 °C. Samples were extracted with water (wt g/vol) and deproteinized overnight at 4 °C with the addition of phosphotungstic acid (10%, Sigma). Concentrations of SCFAs were determined using a gas chromatograph (GC; Agilent 6890N Network) equipped with a split-splitless injector (GC Agilent 7890B), a flame-ionization detector and a capillary column (15 m × 0.53 mm × 0.5 µm) packed with SP 1000 (Nukol; Supelco 25,236). The flow rate of hydrogen, the carrier gas, was 10 mL/min; the temperatures of the injector, column and detector were 200 °C, 100 °C and 240 °C, respectively. 2-Ethylbutyrate was used as the internal standard. Two replicates were performed for each sample. We collected the SCFA data and integrated the peaks using the GC’s default software (Agilent). To determine the final concentrations of SCFAs, the samples were weighed before and after protein precipitation to obtain the appropriate multiplication factor.

### Ussing chamber experiments

Paracellular pathway permeability was measured using the flow of TRITC-dextran 4KD (TD4), fluorescein sulphonic acid (FSA) and horseradish peroxidase (HPR) through colon and ileum samples, which were opened along the mesenteric border and mounted in Ussing chambers (P2300, Physiologic Instruments, USA). At 37 °C, 0.2 cm^2^ of tissue surface was exposed to 2.5 ml of 10-mM oxygenated Krebs-glucose and 10 mM Krebs-mannitol (serosal and luminal sides, respectively). TD4, FSA and HPR (0.4 mg/ml) were added to the mucosal chamber, and samples were collected from the serosae chamber every 15 min for 2 h. TD4 and FSA concentrations were determined using a microplate reader (Tecan). HRP concentrations were determined by an enzymatic reaction using the substrate o-dianisidine as previously described. Transepithelial conductance was measured by clamping the voltage and recording the change in the short-circuit current (Isc). At the end of the experiment, tissues were challenged with the cholinergic analogue carbachol (CCh) on the serosal side (100 mM), and the *Δ*Isc was recorded to check the viability of the tissue.

### Microbial DNA extraction and 16S sequencing analysis

Genomic DNA was extracted from mice’s colon (at 5 days), colon content (at endpoint), faeces (the rest of the time points) and mothers’ faeces, skin and vaginal washings using the mouse Stool Mini Kit (QIAGEN, Germany), according to the manufacturer’s instructions.

The V3–V4 region of the 16S RNA gene was amplified with PCR1F_343 (5′-CTTTCCCTACACGACGCTCTTCCGATCTACGGRAGGCAGCAG-3′) and PCR1R_784 (5′-GGAGTTCAGACGTGTGCTCTTCCGATCTTACCAGGGTATCTAATCCT-3′) primers, and sequencing was performed on an Illumina MiSeq platform (Illumina, USA). The 16S RNA raw reads are available at NCBI’s SRA repository (Bioproject PRJNA876103; accessions SAMN30638467 to SAMN30638852).

Quality control was performed on the resulting FastQ files using FastQC software (https://www.bioinformatics.babraham.ac.uk/projects/fastqc), and bioinformatics analyses were conducted by using the Quantitative Insights into Microbial Ecology (QIIME 1.9.1) software package [33] with the subsampled open-reference OTU picking approach [34] and the Greengenes reference database (version 13.8) [35]. Singleton OTUs and OTUs representing less than 0.005% of the total number of sequences were removed from the dataset as suggested by the software developers [36]. Chimeric sequences were identified using the BLAST algorithm and removed using QIIME.

Microbiota diversity analyses were conducted in R by using the phyloseq package (v. 1.22.3) [37]. Richness and diversity analyses were performed at the OTU level. Alpha diversity and beta diversity were calculated using the Shannon index and Whittaker’s index, respectively. Richness was defined as the total number of OTUs present in each sample. The vegan (v. 2.5–3) package was used to perform nonmetric multidimensional scaling (NMDS) by using Bray–Curtis dissimilarity values. The *env_fit* function was used to evaluate the statistical significance of the study variables within NMDS ordination space. In addition, permutational multivariate analyses of variance were performed using distance matrices and the *adonis* function (alpha level, *p* < 0.05). The OTU differential abundance testing was carried out with the metagenomeSeq package (v1.20.1) [38] with OTU counts normalized using the cumulative sum scaling (CSS) method and a zero-inflated Gaussian distribution mixture model (*fitZig* function). The significance level was set to a false discovery rate (FDR) lower than 0.05.

### Microbial networks inference

Two microbial networks, one for each of the delivery mode (VD and CSD), were inferred at genus level with SPCIT approach [39]. In brief, SPCIT is an ensemble approach that first calculates correlations between features using the Sparse Correlations for Compositional data software [40], and, in a second step, estimate the significant correlations using a data-driven methodology based on partial correlation and information theory [41]. In the network, every node represents a genus, and every edge (connections between nodes) represents a significant correlation. Cytoscape software [42] was used to visualize the network and to calculate the node centrality values and network topological parameters using the CentiScaPe plug-in [43].

### Metagenome functional predictions

The bioinformatics software package PICRUSt [44] was subsequently used to predict the functional content of the inferred synthetic metagenomes. Samples clustering was performed on R (version 3.6.1, https://www.r-project.org/) according to the KEGG Orthology (KOs) abundance profiles, based on Euclidean distance measure as metric and Ward-linkage method. The principal coordinates analysis (PCoA) of KOs Euclidean distances was plotted using the R vegan package [45]. After PICRUSt prediction, we collected from the 6.909 KOs the ones mapping into the butyrate syntheses pathways. Three KOs (butyrate kinase (buk, K00929), vinylacetyl-CoA 3,2-isomerase (AbfD, 4Hbt, K14534) and butyryl-CoA:acetate CoA transferase (Ato, K01034)) were recovered, and the mean abundance was compared at each point (Wilcoxon rank-sum test) between VD and CSD groups. The same mean comparison between groups was performed at genus level, considering only from the whole dataset only bacterial genera described as butyrate producers (*Odoribacter*, *Coprococcus*, *Roseburia*, *Anaerostipes* and *Clostridium*) [46].

### Mouse gene expression and microarray analysis

Total RNA was isolated from the colon samples using RNeasy Mini Kit (QIAGEN), according to manufacturer’s instructions. The RNA integrity was verified in Bioanalyzer 2100 with RNA 6000 Nano Chips (Agilent Technologies). Only samples with RNA integrity > 9 were selected for the study. Mouse transcriptomics was performed using SurePrint G3 Mouse GE 8 × 60 K Microarray (Design ID: 028005, Agilent Technologies), according to manufacturer’s instructions in the Genomic Facility and aBridge platform of UMR 1313 GABI. Microarray data are deposited in GEO under the accession number GSE214311. Probe intensities were corrected, log_2_ scaled and quantile normalized using the limma R package version 3.38 [47]. In order to remove noise from expression patterns associated with very weakly expressed genes, the probes with values higher than the lowest 5% quantile of each sample, in at least 4 samples, were retained. Other probes were filtered out, and controls were discarded. Subsequently, we averaged expression of probes representing the same gene. To identify the differential expressed genes between CSD and VD samples, we fit a linear model for each gene using the eBayes function from the limma R package, setting the sex and the delivery mode as fixed effects. The biological mother factor was included as a random effect using the “duplicateCorrelation” function included in the limma package. Significance was declared after adjusting raw *p*‐values to control the false discovery rate (FDR) at 5% using the Benjamini–Hochberg procedure [48]. Selected gene lists (log ratio and *p*-value data) were loaded into Ingenuity Pathway Analysis to analyse pathways and generate data displays.

### Statistical analyses of mouse phenotypes

Statistical analyses were performed using GraphPad v9 (GraphPad software, San Diego, CA, USA). Survival curve analyses have been performed by logrank test (Mantel Cox). For weight curves, a multiple unpaired *T*-test was performed per day with fewer assumptions corrected for multiple comparison with Holm-Sidak method. Normality and variance analysis were performed using Shapiro–Wilk normality test and one-way ANOVA (Brown-Forsythe test), respectively. For normal samples (Gaussian distribution) with equal variances, three-way ANOVA has been performed to compare the effect of the delivery, treatment and sex for the DNBS trials; multiple comparisons were carried out using Tukey’s test. For non-normal samples or/and with unequal variances, nonparametric tests have been performed (Kruskal–Wallis test). Multiple comparisons were carried out using Dunn’s test. *p*-Values < 0.05 were considered statistically significant.

## Results

### CSD increases susceptibility to chemically induced inflammatory insults at weaning and adulthood

**Fig. 1:**
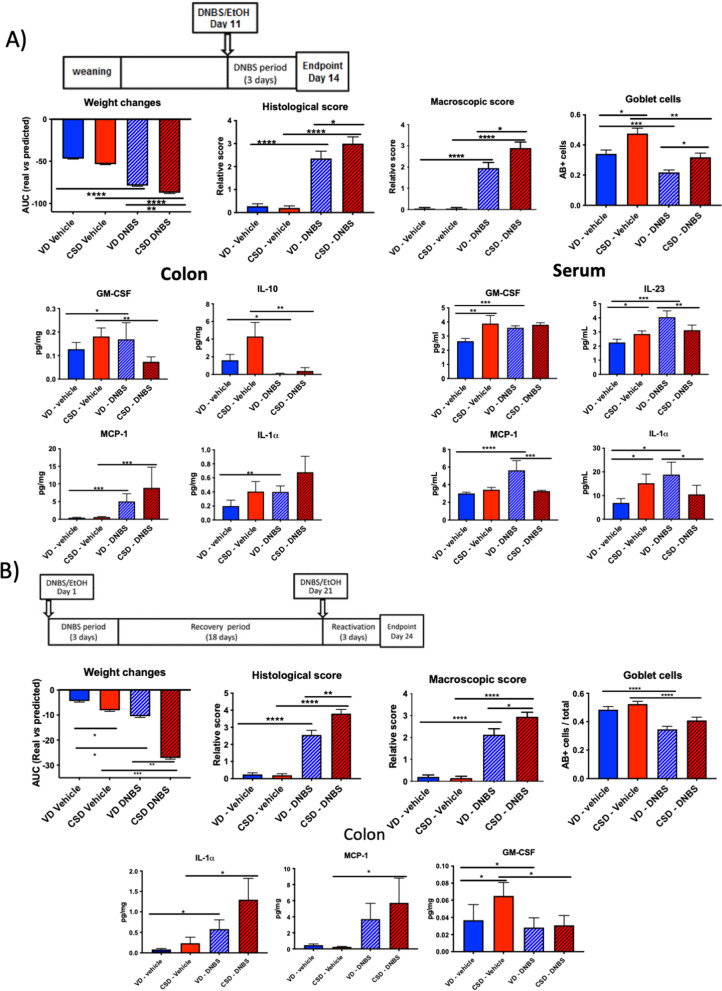
Acute and chronic chemical-induced colitis experiments. **A** DNBS-induced acute colitis model protocol. **B** DNBS-induced chronic colitis model protocol, weigh changes after DNBS injection, histological score, macroscopic score, goblet cells percentages, colon cytokine levels and serum cytokine levels. Groups: vaginal-delivered mice, non-inflamed (VD, vehicle, blue solid); vaginal-delivered mice, inflamed (VD-DNBS, blue striped); C-section-delivered mice, non-inflamed (CSD, vehicle, red solid); C-section-delivered mice, inflamed (CSD-DNBS, red striped). *n* = 20. DNBS, 2,4-dinitrobenzene sulphonic acid; EtOH, ethanol; GM-CSF, granulocyte–macrophage colony-stimulating factor; MCP-1, monocyte chemoattractant protein-1. *p-value < 0.05; **p-value < 0.01; ***p-value < 0.001; ****p-value < 0.0001

As CSD has been associated with an increased risk of CD [27], we submitted CSD and VD mice to two chemically induced colitis models: (i) an acute colitis protocol soon after weaning to mimic a first inflammatory flare in CD patients (Fig. 1A) and (ii) a chronic colitis protocol in young adulthood (6 weeks) to mimic the relapse and crisis cycles observed in CD patients (Fig. 1B). In both cases, CSD mice were more susceptible to the induced inflammatory challenge in terms of weight changes, histological, macroscopic scores and GC depletion (typical parameters of DNBS-induced colitis, Fig. 1A–B). Furthermore, mice delivered by CSD showed altered colon and serum cytokine profiles (GM-CSF, IL-23, MPC-1 and IL-1α) in the acute protocol (Fig. 1A) and colon profiles (Il-1α, MCP-1 and GM-CSF) in the chronic protocol (Fig. 1B). These results point toward a significant increase in the susceptibility to induced intestinal inflammation of CSD mice as shown by increased clinical signs of disease and inflammatory parameters. These results are in line with clinical data showing that infants born through CSD have a higher risk of developing IBD and with previous studies linking CSD with increased susceptibility to induced inflammation in mice [27, 49].

### CSD mice are colonized by a more complex microbiota in the very early life

**Fig. 2:**
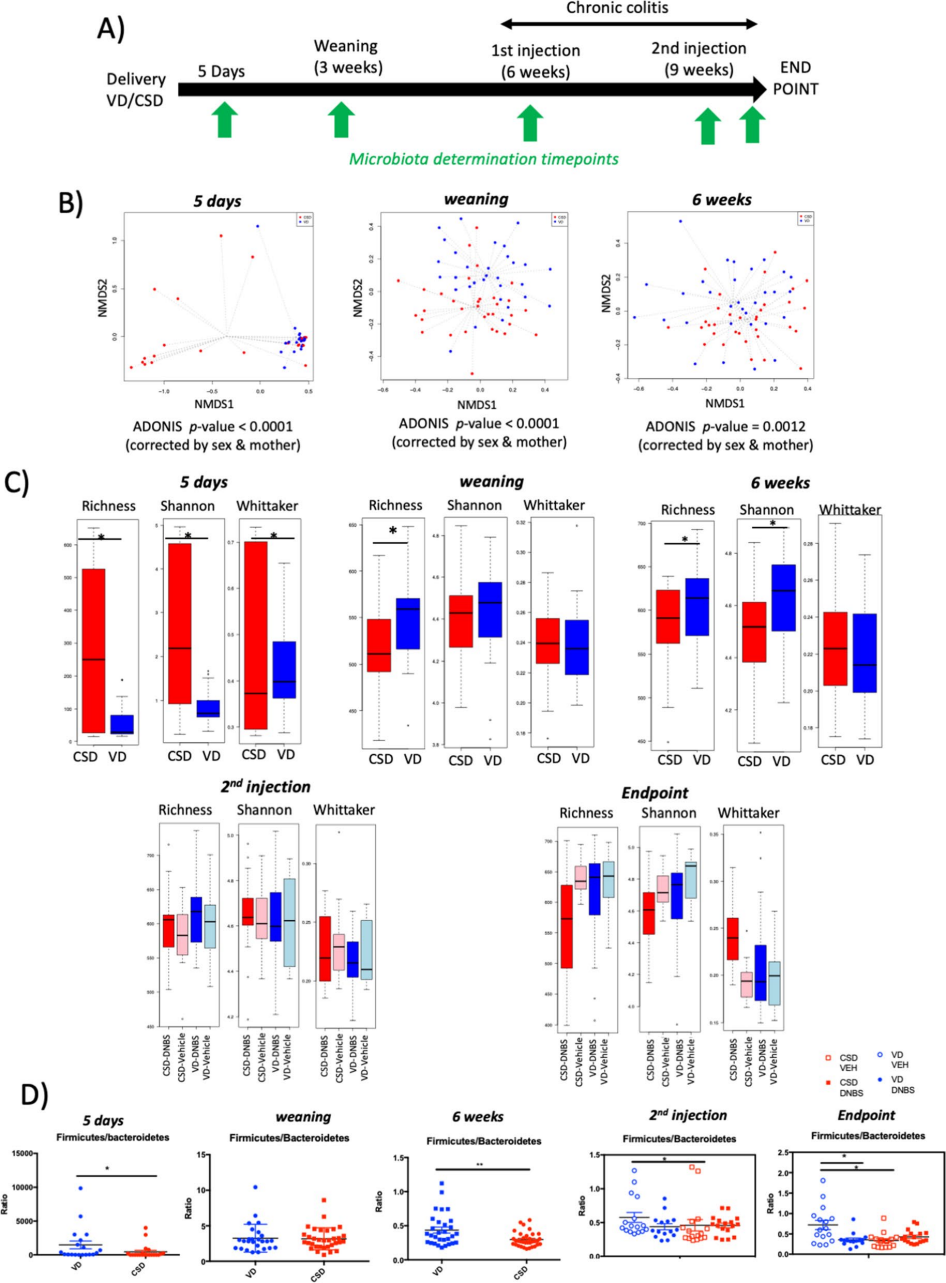
Microbiota analysis in early life. **A** Schema of the sampling procedures. **B** Nonmetric multidimensional scaling (NMDS) analyses based on the Bray–Curtis. **C** Richness, alpha and beta diversities and richness (measured by Shannon and Whitaker index). **D** *Firmicutes/Bacteroidetes* ratios. Sample size: 5 days, *n* = 31–33; weaning *n* = 31–33; 6 weeks, *n* = 32; second injection and endpoint, *n* = 18. Groups at 5 days, weaning and 6 weeks: vaginal delivery (VD, blue) and C-section delivery (CSD, red). Groups at second injection and endpoint: vaginal-delivered mice, non-inflamed (VD, vehicle, light blue); vaginal-delivered mice, inflamed (VD-DNBS, dark blue); C-section-delivered mice, non-inflamed (CSD, vehicle, light red); C-section-delivered mice, inflamed (CSD-DNBS, dark red). *p-value < 0.05; **p-value < 0.01

In both humans and mice, CSD has been related to gut microbiota modifications. We thus compared the gut microbiota evolution at different time points between CSD and VD pups from 5 days until the end of the chronic colitis protocol (Fig. 2A). In general, 5-day microbiota was very different from the other time points tested (Fig. S1). Nonmetric multidimensional scaling (NMDS) analyses based on the Bray–Curtis dissimilarities showed that the microbiota composition was different according to the delivery method at 5 days, weaning and 6 weeks (Fig. 2B; *p*-value < 0.0001). Compared to CSD, VD pups have a decreased alpha diversity (Shannon index) and richness at 5 days (*p*-value < 0.05; Fig. 2C). In contrast, richness at weaning and 6 weeks and alpha diversity at 6 weeks were significantly higher in VD pups versus CSD ones (*p*-value < 0.05, Fig. 2C). Regarding the beta diversity, which concerns the differences within the groups, the Whittaker index was increased in CSD pups at 5 days (*p*-value < 0.05), indicating that microbiota is more heterogeneous within the CSD group compared to VD group. This shift from higher diversity to a lower alpha diversity in CSD individuals compared with VD ones and a higher heterogenicity among CSD microbiotas has also been observed in humans [19]. At 5 days and 6 weeks, composition differences were observed (*p*-value < 0.05, Tables S1, S2 and S3, Fig. S2A–B). The most affected genus was *Lactobacillus* which was reduced in CSD pups at 5 days (Table S4). In addition, CSD pups showed a decreased *Firmicutes/Bacteroides* ratio, often used as an indicator of bacterial ecosystem status (Fig. 2D). This result is not concordant with the microbiota differences found in humans, where CSD decrease the abundance of *Bacteroidetes* [18, 20]. These differences between mice and human responses to CSD in microbiota composition could be due to observed differences in genus-/species-specific abundances between the murine and the human gut microbiota that mirror the inherent differences between these two mammalian systems and their lifestyles [50].

Regarding the chronic colitis protocol, the differences between VD and CSD disappear before the second injection and endpoint (data not shown). However, CSD pups react stronger to DNBS, with a higher decrease in alpha diversity and richness and a stronger increase in beta diversity at the endpoint (Fig. 2C), underlining a loss of bacterial diversity and a higher sample heterogenicity in CSD group in response to DNBS. Compositional differences were also observed (Fig. S2C–D). Of note, *Firmicutes/Bacteroides* ratio is decreased in non-inflamed CSD mice compared to non-inflamed VD mice, while CSD mice (inflamed or not) were similar to VD-inflamed mice (Fig. 2D).

Taken together, these results suggest that CSD pups, in contrast to VD ones, suffer a faster complexified primary colonization, show a higher diversity at birth followed by a slower expansion thereafter as it has been described in humans [51] and react stronger to colitis by losing microbiota diversity.

### CSD pups show a particular microbial correlation pattern in early life

**Fig. 3:**
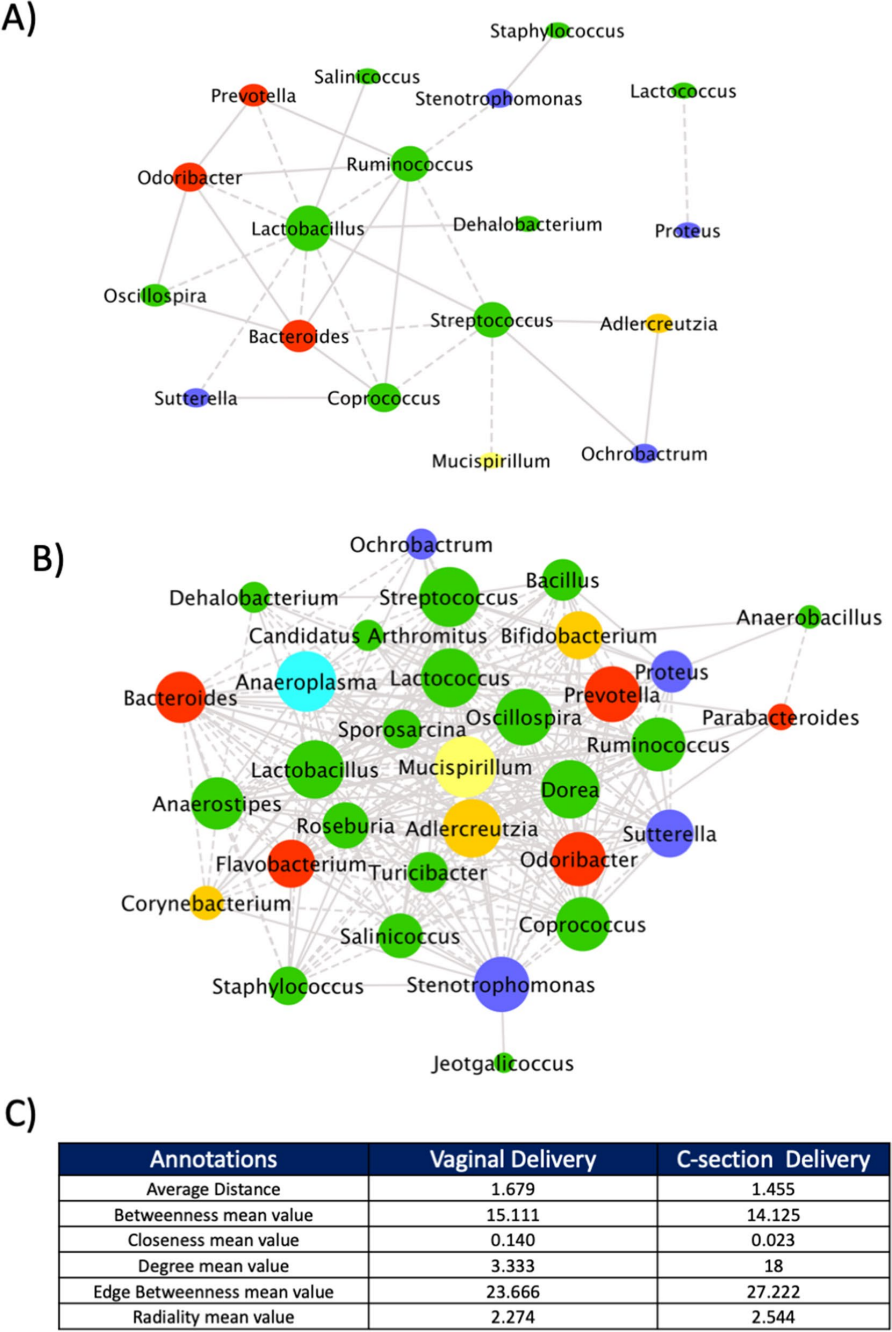
Microbiota origin and network. **A** and **B** Typical bacterial hub of VD (**B**) and CSD (**C**) mice at weaning. **C** Main parameters of VD and CSD pup networks. *N* = 31–33

Bacterial genera which were present in > 20% of the animals at 5 days (*n* = 34) were employed to infer the microbial correlation network using the SPCIT approach [39]. The resulting microbial network led to the identification of 18 nodes and 30 edges for VD pups and 32 nodes and 288 edges for CSD (Fig. 3B–D). As expected, in VD pups, the network centrality parameters indicate *Lactobacillus*, a dominant bacterium of the murine gut and vaginal microbiota as keystone genera (central node with the higher number of correlations) in the VD network (Fig. 3B). Instead, *Mucispirillum*, which inhabits the mucus layer, was identified as the keystone genus in samples collected from CSD mice (Fig. 3C).

These results indicate the presence of a completely different bacterial ecosystem due to the delivery method with potential consequences for the host. Although CSD pups display an excessive mature microbiota in their early life, they put aside key members of the gut microbiota as it is the case of *Lactobacillus* in mice, a major member of the murine gut microbiota. Taken together, these results highlight the presence of a complex ecosystem in CSD pups in contrast to VD ones whose gut ecosystem is still primordial. Nevertheless, caution should be made in transferring these findings to the human context as the murine and human gut microbiota differ in composition [50]. Besides, network analysis performed using a single time point may have missed potential additional correlations due to a limited sample size and lack of power.

### CSD pups’ microbiota has altered functionality and SCFAs production capacities

To gain insight into the peculiar functional variations of the gut microbiota, correspondent metagenomes were inferred using PICRUSt [44]. Clustering analysis highlighted a clear separation according to delivery mode (*p*-value = 2 × 10^−4^) (Figs. S3 and 4). In particular, the microbiota of CSD pups showed the presence of a higher metabolic activity than VD pups at 5 days (976 KEGG orthologs (KOs) overabundant and 143 underabundant in CSD versus VD pups). The differences between CSD and VD mice were maintained at 5 days (*p*-value < 0.0001), weaning (*p*-value = 0.2) and 6 weeks (*p*-value < 0.0001), along the inflammation protocol and until the endpoint in the vehicle groups (*p*-value < 0.0001).

**Fig. 4:**
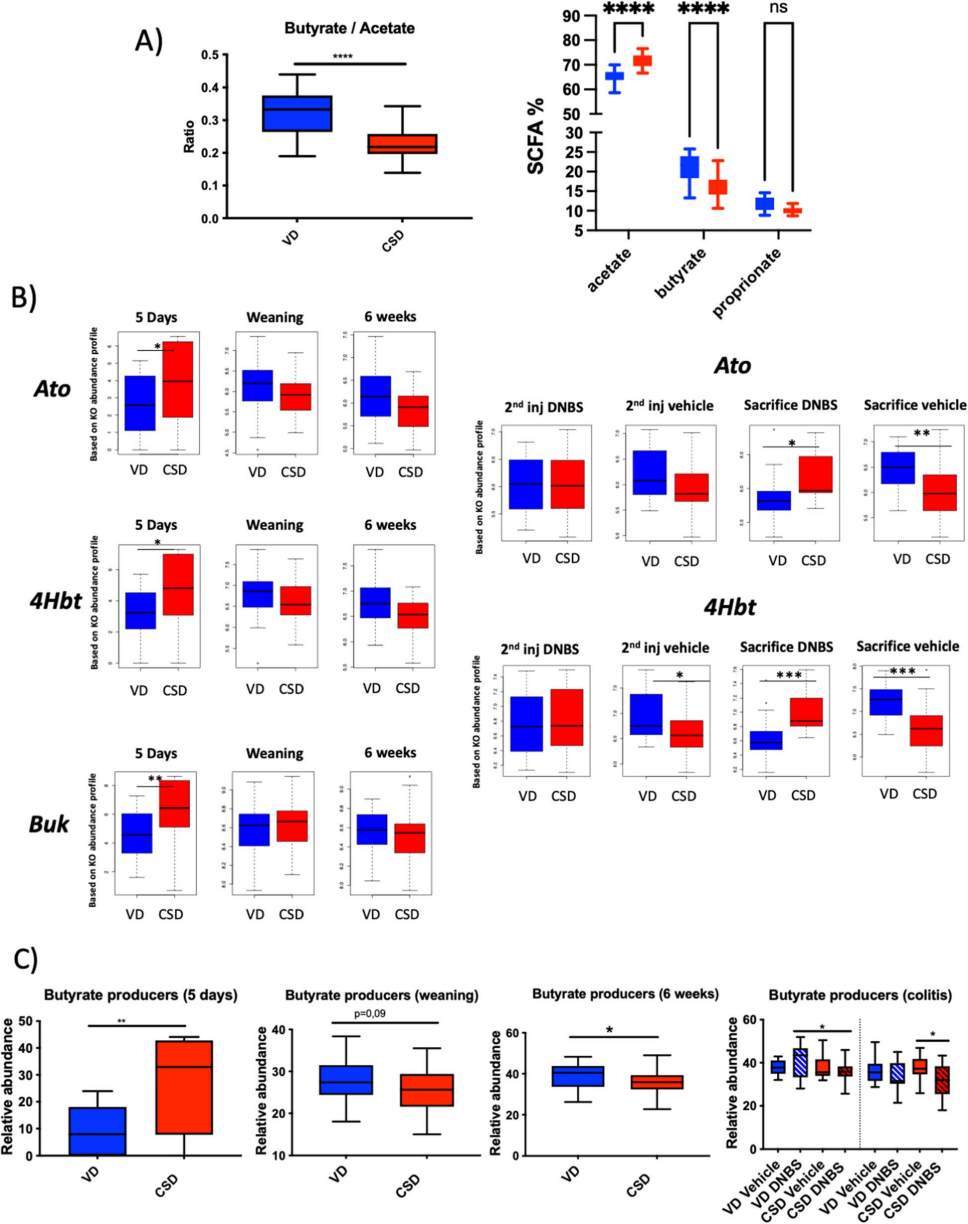
Short-chain fatty acid analyses. **A** Butyrate/acetate ratio and percentage of acetate and butyrate at caecum samples at weaning. **B** Modulation of KEGG orthologs involved in butyrate production pathway predicted by PICRUSt approach (butyrate kinase [buk, K00929], vinylacetyl-CoA 3,2-isomerase [AbfD, 4Hbt, K14534] and butyryl-CoA:acetate CoA transferase [Ato, K01034]). **C** Comparison of butyrate producer genus at 5 days, weaning, 6 weeks, second injection and endpoint (VD versus CSD). Groups: vaginal-delivered mice, non-inflamed (VD-vehicle, blue solid); vaginal-delivered mice, inflamed (VD-DNBS, blue striped); C-section-delivered mice, non-inflamed (CSD vehicle, red solid); C-section-delivered mice, inflamed (CSD-DNBS, red striped). *N* = 18–22. *p-value < 0.05; **p-value < 0.01; ***p-value < 0.001; ****p-value < 0.0001

As SCFAs are one of the most important metabolites produced by the gut microbiota, we determined their concentration in caecum samples at weaning. CSD exhibited a lower butyrate/acetate ratio due to both an increase in acetate and a decrease in butyrate proportion (*p*-value < 0.05) (Fig. 4A), highlighting a possible dysregulation of the metabolic interdependence between these two SCFAs. Indeed, at 5 days, CSD pups presented an increase in the presence of the main activities related to butyrate synthesis (buk, 4Hbt and Ato) (*p*-value < 0.05). At weaning, 4Hbt and Ato tended to be decreased (Fig. 4B). Of note, Ato is the enzyme that converts acetate to butyrate, confirming the potential dysregulation of this process. During inflammation, Ato and 4Hbt were increased in CSD compared to VD in non-inflamed groups and decreased in inflamed ones (Fig. 4B).

Subsequently, we analysed the presence of butyrate-producing genera (Fig. 4C). Only 5 butyrate-producing bacterial genera [46] were detected in our samples (*Odoribacter*, *Coprococcus*, *Roseburia*, *Clostridium and Anaerostipes*) (Table S5). Their abundance in CSD pups increased at day 5 and decreased at later time points (Fig. 4C), in accordance with PiCRUSt data and SCFA dosages. At the second injection, the butyrate producer genera relative abundances were significantly lower in DNBS-inflamed CSD pups than in VD-inflamed ones (Fig. 4C), with a significant reduction of one butyrate producer genera (*Anaerostipes*) (*p*-value < 0.05). This tendency continues at the endpoint with a significant reduction in butyrate producer levels when comparing CSD-vehicle mice to CSD-DNBS mice (*p*-value < 0.05).

Taken together, these findings suggest that SCFA production is altered by CSD in early life, switching from an increase in butyrate producers just after birth, to a decrease of their abundance thereafter. Of note, CSD human infants have more butyrate in faecal samples than VD ones in the early life [52]. This suggests the presence of an overly mature bacterial microbiota early in life and the consequent dysregulation of intestinal homeostasis from this moment onward. Furthermore, DNBS insult allowed us to detect the altered reaction of CSD mice microbiota to the inflammatory challenge, with a decrease in the levels of butyrate producers. SCFAs stimulate mucin synthesis, increase cell proliferation, modulate enterocyte differentiation and have immunomodulatory effect [53–55]. Due to the importance of SCFAs on gut homeostasis, our results point to butyrate and acetate disturbances as the potential microbial precursors of the increased sensitivity to inflammation observed in CSD pups in the long term.

### CSD alters gut barrier structure and permeability

**Fig. 5:**
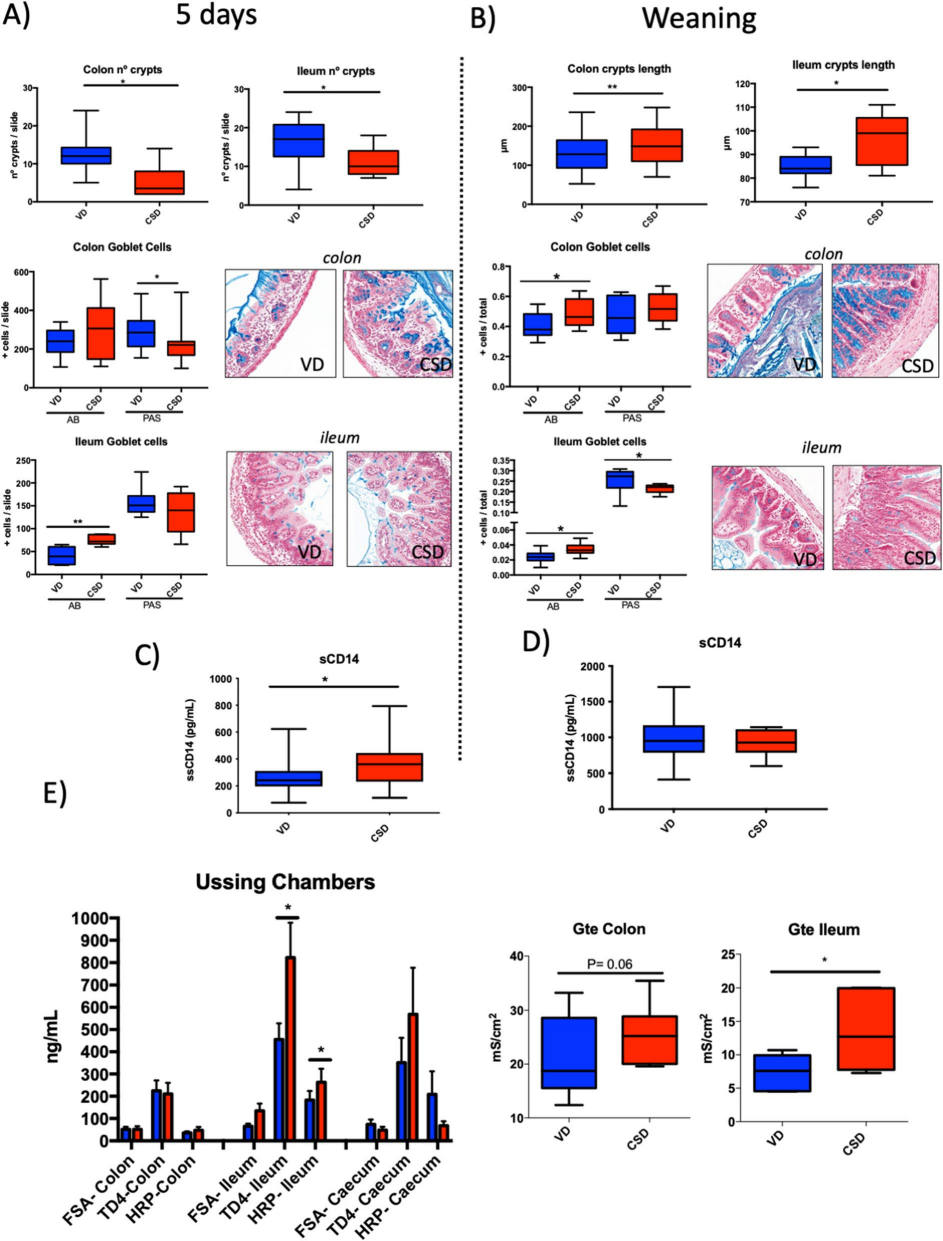
Gut barrier structure and permeability features at 5 days and weaning. **A** Colon or ileum number of crypts at 5 days (*n* = 7–8); percentage of goblet cells along with representative photos of colon and ileum samples, stained by Alcian blue or PAS. **B** Crypt length and goblet cell percentages along with representative photos of colon or ileum samples, stained by AB or PAS at weaning (*n* = 6–10). **C** and **D** Concentration of sCD14 in serum samples at 5 days (*n* = 20) and weaning (*n* = 20). **E** Global permeability measured by the tracer FITC-dextran in serum at weaning (*n* = 28). Permeability to the tracers FSA, TD4 and HRP of colon, ileum and caecum tissues mounted in Ussing chambers (*n* = 10). Electrical conductance of colon and ileum tissues mounted in Ussing chambers (*n* = 10). Groups: vaginal delivery (VD, blue) and C-section delivery (CSD, red). AB, Alcian blue; PAS, periodic acid-Schiff. *p-value < 0.05; **p-value < 0.01

Since the microbiota has been found to participate in gut barrier maturation [56], we decide to evaluate the physical intestinal barrier by analysing the epithelial structure and population of GC, responsible for mucus production, in the colon and ileum. In mice, adult-type epithelial enterocytes appear around 2 weeks of age, and complete closure of the gut barrier occurs around weaning [57]. HES-stained slides showed that CSD pups have lower crypts density at 5 days and longer crypts at weaning (*p*-value < 0.05; Fig. 5A, B). CSD pups have significantly higher Alcian blue (AB, acid mucin specific)-positive GCs at both 5 days and weaning (*p*-value < 0.05), and less periodic acid-Schiff (PAS, acid and neutral mucin staining) GCs at 5 days, pointing out for an increase of acidic mucin-producing GCs and a decrease in neutral ones due to CSD (Fig. 5A, B).

Since the selective permeability of the intestinal barrier is very important in the early life [56], we determined the effect of these structural differences on global permeability. At 5 days, CSD pups showed higher levels of sCD14, a marker for bacterial translocation (Fig. 5C). However, these differences were corrected at weaning (Fig. 5D). To directly assess local permeability at weaning, colon, ileum and caecum samples from CSD and VD, mice were mounted in vitro in Ussing chambers. CSD pups showed increased paracellular (TRITC-Dextran, TD-4) and transcellular (Horseradish peroxidase, HRP) ileum permeability, but no change in colon and caecum were detected (Fig. 5E). Besides, electrical measures showed an increased conductance in CSD ileum (*p*-value < 0.05) and a trend in colon tissues (*p*-value = 0.06, Fig. 5E) confirming the presence of altered tissues at both colon and ileum.

Taken together, these data showed that both colon and ileum of CSD pups exhibited an increase in acid mucins, as observed in chronically inflamed epithelium in humans [58] and an altered acid/neutral mucin ratio as observed in IBD patients [59]. Furthermore, the epithelial structure was modified due to CSD, with an increase in crypt numbers or size with the consequent increase on intestinal permeability, a feature commonly observed in a low-grade intestinal inflammation.

### CSD modulates host transcriptome, especially functions related to intestinal morphology

**Fig. 6:**
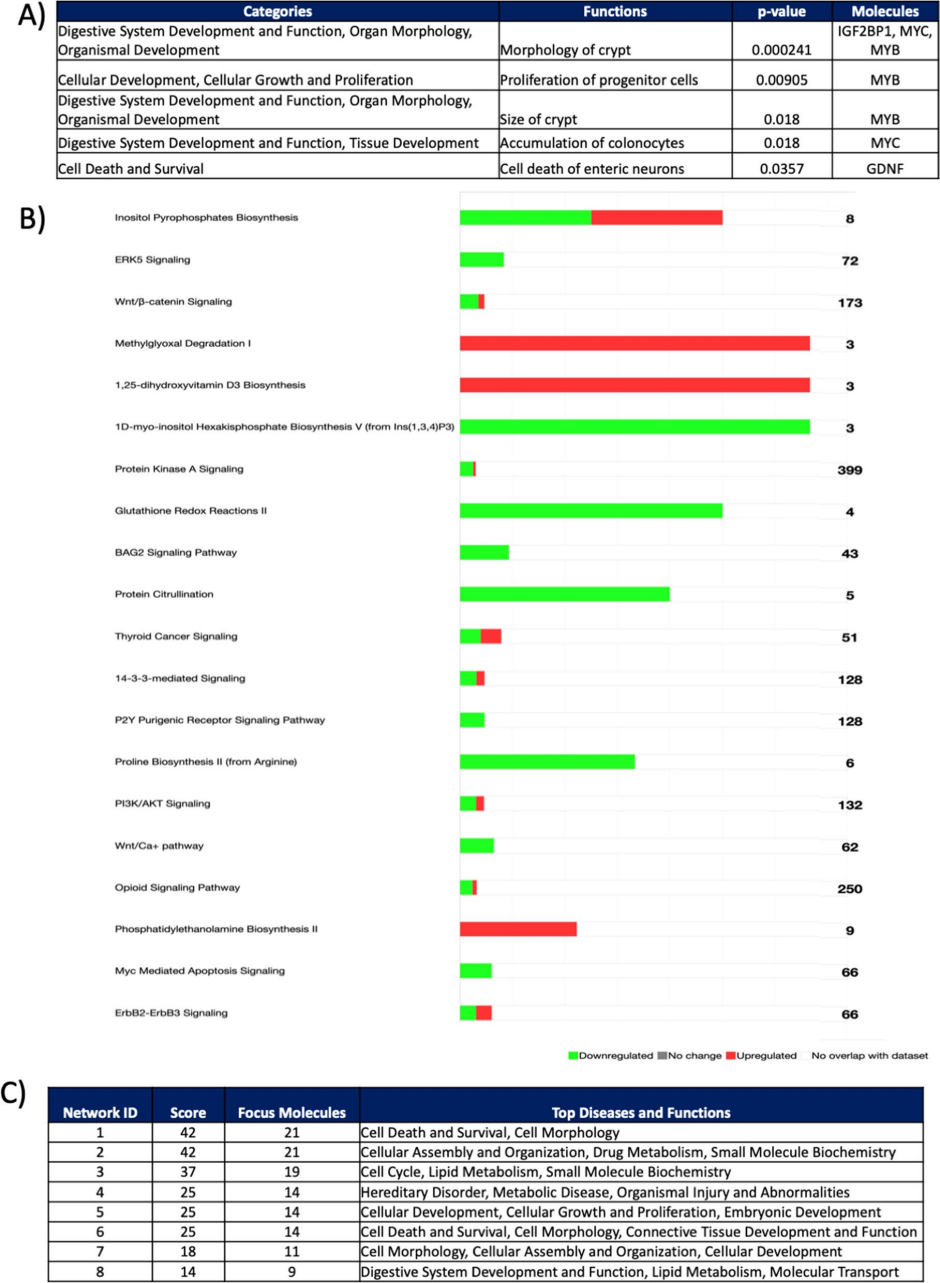
Transcriptome analysis of colon samples at weaning. A Functional analysis of differential expressed (DE) genes (*n* = 5). B Predicted Effect (*p*-value < 0.005) of DE genes on biological functions modulated by CSD and related categories. Top ranked canonical pathways (−log(*p*-value) > 1.3) in Ingenuity Pathway Analysis (IPA) for DE genes between CSD and VD pups (green downregulated, red upregulated). C Top-ranked enriched networks based on IPA network analysis of differentially expressed proteins and metabolites in the CSD group and diseases and functions related

To explore the mechanisms underlying the effects of CSD on gut barrier structure and immunomodulation, we analysed the tissue colon transcriptome of CSD and VD pups at weaning using microarray Agilent Technology. Transcriptome analysis revealed that the expression of 178 genes differed between the two groups (adj. *p*-value < 0.05) (87 upregulated and 91 down-regulated genes; Table S6). In line with the observed structural abnormalities, CSD pups exhibited an increased expression of genes related to cell survival, progenitor cells proliferation and colonocytes accumulation at weaning (*IGF2BP1*, *MYC*, *MYB*, *GDNF*; Fig. 6A). Functional analysis of all the modulated genes showed that 19 pathways were differentially modulated by CSD (log *p*-value < 1.3; Fig. 6B). Among them, we found protein kinase A signaling and PI3-kinase/Akt pathways as well as Bcl2-associated athanogene, all of which related to abnormalities in proliferation rates [60–63]. Furthermore, CSD was associated with alterations in several pathways related to cellular responses to stress, signaling and cell differentiation including ERK5, 14–3-3 and inositol pyrophosphate pathways [64–66]. Besides, CSD was also associated with alterations in Wnt/β-catenin and Wnt/Ca^+^ signalling; both pathways involved in the maintenance of stem cells and differentiation into specific cell lineages such as secretory cells in the intestine [67].

The IPA upstream regulators analysis that in silico identifies the upstream regulators that may be responsible for gene expression changes showed significant activation of the regulator mir-21, an onco-miR which acts by inhibiting the expression of phosphatases limiting AKT and MAPK signaling and unchaining uncontrolled proliferation [68]. Finally, network analysis using IPA revealed the presence of 8 CSD-modulated networks (Fig. 6C), including several networks related to cellular morphology, assembly, growth and proliferation, digestive system development and lipid metabolism.

Taken together, the transcriptome results confirm that CSD is associated with an alteration of a network of genes involved in cell death, survival and proliferation, leading to an altered intestinal morphology at weaning.

### CSD modify local and systemic immune status

**Fig. 7:**
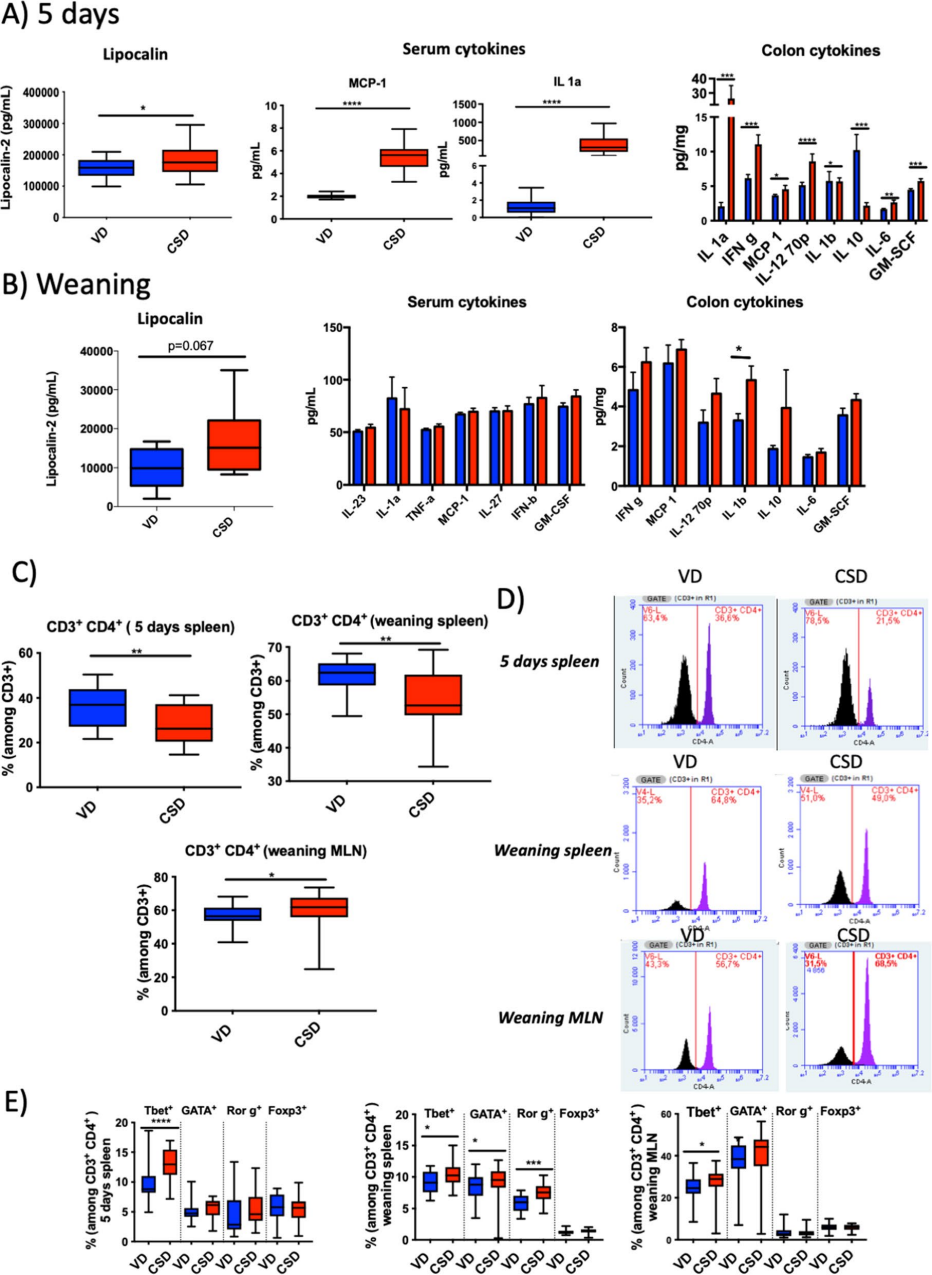
Local and systemic immunity parameters. General inflammatory parameters at 5 days (**A**) and weaning (**B**). Lipocalin-2 concentration in serum at 5 days (*n* = 19–21) and weaning (*n* = 10); cytokine concentrations in serum, colon and ileum (*n* = 10). **C** Positive CD3^+^/CD4.^+^ cells detected by flow cytometry in spleen at 5 days and mesenteric lymph nodes (MLN) and spleen at weaning (*n* = 20) and **D** representative flow charts. **E** Positive Th cells detected by flow cytometry in spleen at 5 days and MLN and spleen at weaning (*n* = 20). Groups: vaginal delivery (VD, blue) and C-section delivery (CSD, red). *p-value < 0.05; **p-value < 0.01; ***p-value < 0.001; ****p-value < 0.0001

In early life, the immaturity of the intestinal barrier function parallels the immaturity of the immune system, resulting in a higher passage of antigens through the intestine [57]. Disruption of the gut barrier could increase mucosal inflammation and even lead to systemic inflammation. At 5 days, CSD pups showed higher serum levels of lipocalin-2 (Lcn-2), an early inflammatory marker (*p*-value < 0.05) (Fig. 7A). The same signal was observed at 3 weeks, although the difference was not statistically significant due to a higher heterogenicity of samples (Fig. 7B). The presence of an early pro-inflammatory status at systemic and intestinal levels in CSD pups was confirmed by the increased levels of pro-inflammatory cytokine levels in serum (MCP-1, IL-1α), ileum (IFN-γ) and colon samples (IL-1α, IFN-γ, MCP-1, IL-6, IL-12 70p, GM-SCF) at 5 days and a decreased level of colonic IL-10 (Fig. 7A). However, at weaning, most of these differences disappeared with the only persistence of an increased IL-1β level in the colon of CSD pups (Fig. 7B).

To further analyse the immune response, major T-cell subpopulations were studied in MLN and spleen. CD3^+^CD4^+^ T-cell percentages among total lymphocytes were higher in VD pups (Fig. 7D). An increase of Th1 (CD3^+^/CD4^+^/Tbet^+^) was observed at 5 days in spleen and in MLN and spleen at weaning in CDS pups (*p*-value < 0.05; Fig. 7E). An increase in Th17 (CD3^+^/CD4^+^/Rorγτ^+^) and Th2 (CD3^+^/CD4^+^/GATA^+^) was also observed in the spleen at weaning (*p*-value < 0.05; Fig. 7E) pointing out for an over activated immune system. No differences were found in regulatory T cells (CD3^+^/CD4^+^/Treg^+^) (Fig. 7E).

Taken together, these results suggest that, in early life, CSD pups display a local and systemic pro-inflammatory context, with fewer and over reactive CD4 T-helper cells. Furthermore, although newborns naturally tend to exhibit a Th-2 skewed immune response to reduce miscarriage [69], IL-12, INF-γ, and CD3^+^/CD4^+^/Tbet increases observed in CSD pups point out a pro-inflammatory Th-1 response linked to CSD. This circumstance might prompt CSD mice to suffer a higher susceptibility to inflammation afterwards.

### Faecal microbiota transplantation from CSD pups at weaning partially transfers the CSD pup phenotypes

**Fig. 8:**
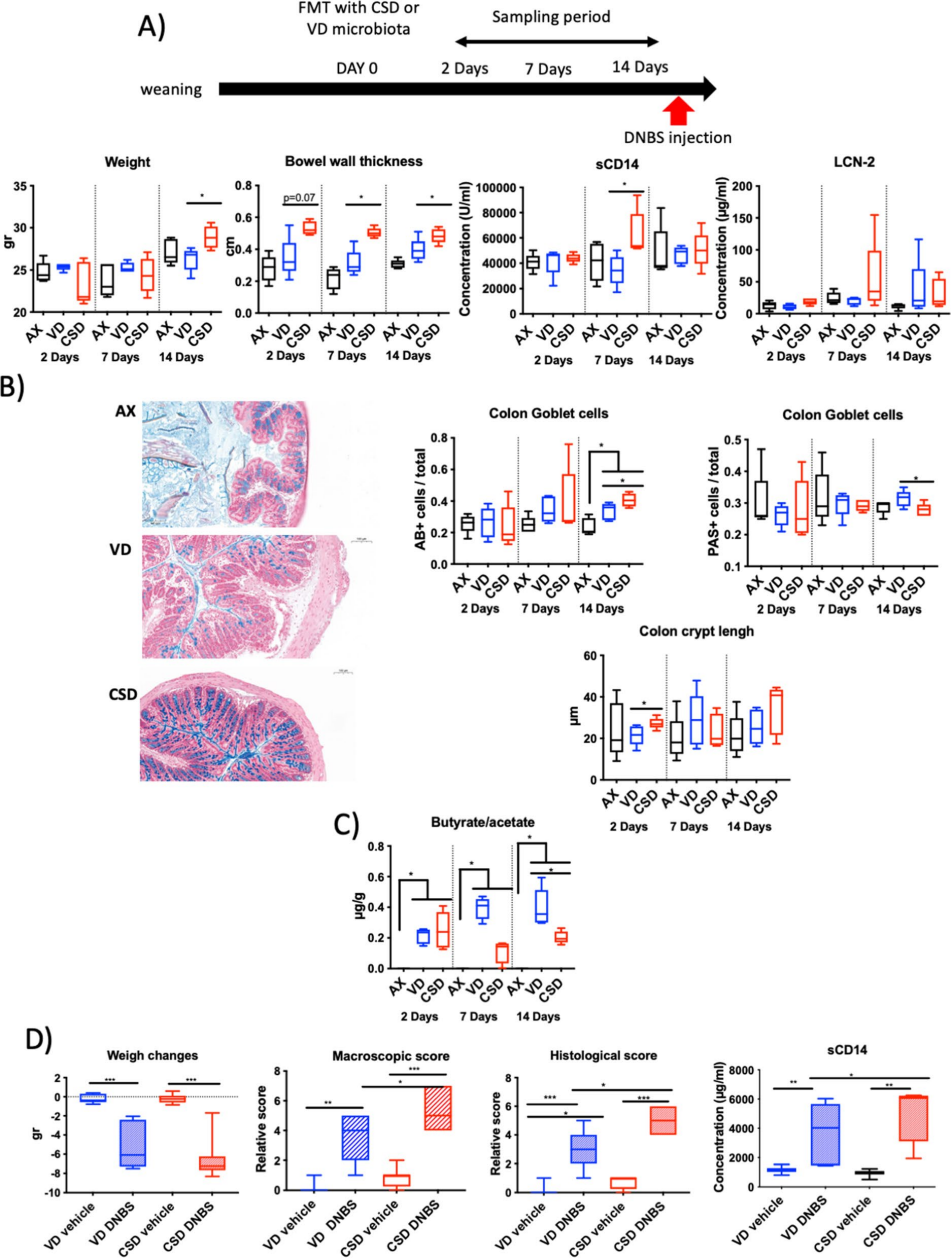
Analysis of ex-germ-free mice colonized with VD and CSD microbiota by faecal microbiota transplantation. **A** Phenotypic parameters at short term: weight, bowel wall thickness, sCD14 on serum samples, lipocalin-2 concentration in serum samples. **B** Histological features representative photos of colon stained with AB; proportion of goblet cells stained with AB or PAS colon crypt length. **C** Butyrate/acetate ratio. **D** Sensitivity to DNBS-induced colitis in the long term: weight changes after DNBS injection, macroscopic score, histological score, global permeability (sCD14 levels). Groups: axenic mice (AX), ex-GF mice colonized with vaginal-delivered faeces (VD, blue = GF-VD), C-section-delivered faeces (CSD, red = GF-CSD). *N* = 5–8. *p-value < 0.05; **p-value < 0.01; ***p-value < 0.001

To directly evaluate the effect of the CSD microbiota on the host without interference with very early-life priming process, 5-week-old GF mice were colonized with faecal samples from CSD and VD mice at weaning (thereafter GF-CSD and GF-VD, respectively) (Fig. 8). Mice were euthanized at different time points after colonization (2, 7, or 14 days) to monitor the effect of the colonization process on the host physiology. At 14 days, an increase in weight gain was observed in GF-CSD as well as an increase in bowel thickness at 7 and 14 days (Fig. 8A). At 14 days, GF-CSD mice showed an increase of acid mucus-producing GCs (GC AB^+^) and a decrease of neutral ones (GC PAS^+^) as it has been observed in CSD pups (Fig. 8B). GF-CSD mice also showed transitory early post-colonization alterations in permeability (measured by sCD14), inflammation (Lcn-2 in serum) (Fig. 8B) and crypt length (Fig. 8B), confirming that some of the modifications observed in GF-CSD are transitory as it has been found in CSD mice. Furthermore, GF-CSD pups showed a reduced butyrate/acetate ratio (Fig. 8C). This result is in line with the one obtained in CSD pups at weaning, time point from which the faecal samples used in the colonization of GF mice came from, and confirms the crucial role of microbiota on SCFA dysregulation on CSD pups.

Two weeks later, the mice were submitted to a DNBS-induced acute colitis protocol. GF-CSD mice were more susceptible to the induced inflammatory insult than GF-VD mice as revealed by a higher macroscopic and microscopic score and a higher intestinal permeability (measured by sCD14) (Fig. 8D). These results confirm the long-term transfer of this phenotype to the GF mice with the microbiota.

Overall, our results indicate major and causal effect of the microbiota on the parameters altered by CSD.

### Supplementation with lactobacilli improves CSD-related phenotype

**Fig. 9:**
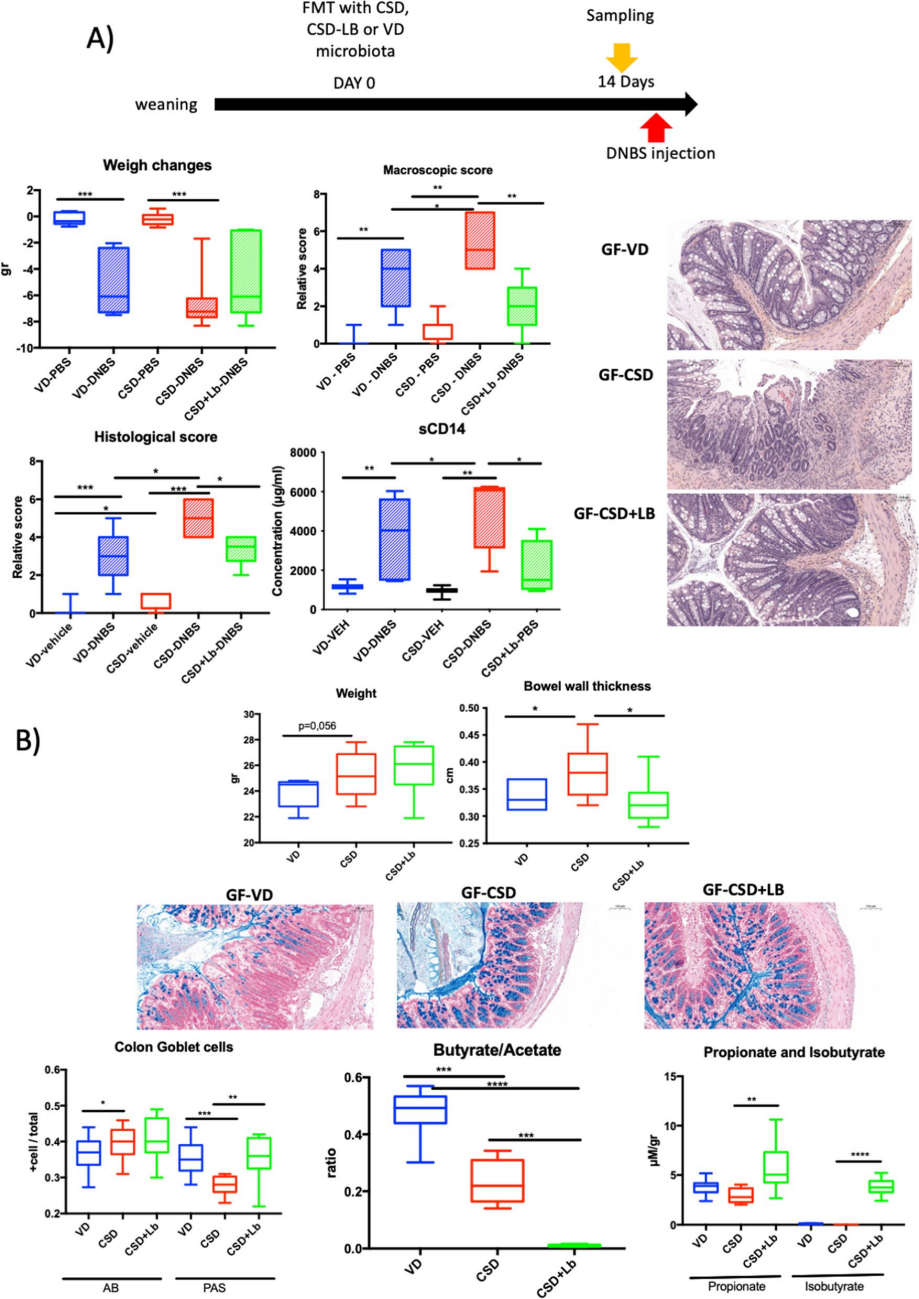
Short- and long-term effects of lactobacilli supplementation in ex-germ-free colonized with CSD microbiota. **A** Sensitivity to DNBS-induced colitis in the long term: weight changes after DNBS injection, macroscopic score, histological score, global permeability measured, thanks to sCD14. **B** Phenotypic parameters at short term: weight, bowel wall thickness, colon AB^+^ or PAS.^+^ goblet cell percentages and representative pictures for AB staining, butyrate/acetate ratio, propionate and isobutyrate concentration on caecum contents. Groups: ex-GF mice colonized with vaginal-delivered faeces (VD, blue = GF-VD); C-section-delivered faeces (CSD, red = GF-CSD); microbiota or C-section-delivered faeces microbiota and lactobacilli (CSD + LB, green = GF-CSD + Lb) subjected (striped, DNBS) or not (solid, vehicle) to DNBS injections. *N* = 5–8. AB, Alcian blue; PAS, periodic acid-Schiff; FMT, faecal material transfer. *p-value < 0.05; **p-value < 0.01; ***p-value < 0.001

As the *Lactobacillus* genus was among the most impacted by CSD and the keystone genera according to our network inference analysis, we decided to supplement GF-CSD pups with a pool of 4 lactobacilli isolated from VD pup’s faecal content at weaning. The lactobacilli were administered to GF mice at the same time than the faecal homogenates from CSD (thereafter GF-CSD + Lb) (Fig. 9A).

GF-CSD + Lb mice were less sensitive to inflammation than GF-CSD, with improved macroscopic, microscopic scores and global permeability (measured by sCD14) (Fig. 9A). These results show the protective role of the early supplementation with lactobacilli regarding CSD-related long-term effects.

Additionally, mice were sacrificed at 14-day post-colonization. The parameters that only varied temporarily on CSD pups (sCD14 and Lcn-2) did not show any modification (data no shown). lactobacilli supplementation did not affect weight changes, bowel wall thickness, or acid GC (GC AB^+^) (Fig. 9B). Nevertheless, lactobacilli increased total GC (GC PAS^+^) to GF-VD levels. These results show that supplementation with lactobacilli partially reverses the effects of CSD before any inflammatory challenge. This effect was associated with a decreased butyrate/acetate ratio and an increased level of propionate and isobutyrate (Fig. 9B). These results suggest that supplementation with lactobacilli compensates butyrate defect by increasing propionate and isobutyrate production which can exert similar beneficial effects [70, 71].

Taken together, our results suggest that supplementing GF-CSD mice with lactobacilli restores most of the epithelial alterations associated with CSD, including susceptibility to colitis in our murine model. Additionally, although GF-CSD + Lb mice still had an altered butyrate/acetate ratio, their ability to increase propionate and isobutyrate levels may be a compensation system as these two SCFA largely share the beneficial effects of butyrate [70, 71].

## Discussion

Recently, the deregulation of symbiotic host-microbe interactions in early life has been shown to cause significant issues related to the maturation of the immune system, predisposing the host to gut barrier dysfunction and inflammation [72]. As CSD babies have an altered gut microbiota [15], these differences might directly affect the development of noncommunicable diseases in later life. In this study, we found that CSD pups were more susceptible to DNBS-induced colitis. The main goal of this study was thus to decipher the role of the early-life gut microbiota alterations linked to CSD on later-life sensitivity to induced intestinal inflammation in mice with a special focus on the gut barrier as the main scenario of the host-microbiota crosstalk.

Several contributing factors are thought to shape the newborn microbiota, including CSD [14, 15]. In our study, CSD pups switched from a higher diversity microbiota at birth to a lower diversity at weaning and at 6 weeks. A correlation network analysis indicated *Lactobacillus* was the keystone (hub genus) of the microbial network of VD pups, while *Mucispirillum* was the main driver of the CSD pups’ gut ecosystem. *Mucispirillum* lives in the mucus layer and is thought to be a pathobiont that plays a role in inflammation.

This increase in diversity in very early life was associated with an alteration in predicted metabolic KO functions, including a switch in butyrate production genes that were increased at 5 days and decreased at weaning. CSD pups showed a higher acetate and a lower butyrate concentration in their caecum content at weaning. Acetate enhances energy production and modulates GC populations [32], a trait observed in our CSD pups. Furthermore, CSD pups are known to grow faster [16], a result confirmed in our study (data not shown) which is in line with the increased acetate concentrations found in CSD pups [73]. Butyrate has immunoregulatory effects on intestinal epithelial cells and regulates multiple functions of gut cells [74]. In our study, butyrate/acetate ratio was altered, similarly as the *Firmicutes*/*Bacteroidetes* ratio.

Maintaining a functional gut barrier is crucial for neonatal development. The physical barrier of the intestinal mucosa is a defence mechanism involving the integrity of intestinal epithelial cells, their associated junctions and the mucus layer [75–77]. The intestinal mucosal layer is mainly composed of mucus but also water, ions and molecules of the immune system such has IgA and AMP which facilitate the clearance of pathogenic microorganisms [78]. Our results indicate that CSD pups have an altered epithelium structure in their early life and a decrease of GC containing neutral mucins, a trait observed in colon inflammation and intestinal infections settings that points to an altered mucus layer [58, 59, 79].

In contrast to restricted macromolecular passage in the adult gut, during the early life, there is an enhanced transfer across the intestine as gut closure appears to occur already in utero in humans and terminates around weaning in mice [57]. In this study, structural gut barrier abnormalities went with an altered permeability. CSD pups passed from an increased systemic permeability at 5 days to restoration at weaning. However, at weaning, an increase in paracellular and mostly in transcellular permeability was measured at the local level in the ileum. The pathway of antigen transfer across the intestinal epithelial barrier could affect the immune system response, being the paracellular passage, the most augmented in this murine model of CSD, responsible for a primary immune response against free antigens and the transcellular passage as educational path for the developing immune system of the newborn [57]. Furthermore, transepithelial electrical measures confirmed the presence of altered tissues in both colon and ileum, with an increased conductance in both tissues. Changes in paracellular permeability are often caused by disassembly of intestinal tight junctions. However, no differences in tight junctions were detected in colonic samples in this study at 5 days or at weaning using transcriptomic approaches (data not shown). Nevertheless, further research should be conducted to fully decipher the host mechanisms underlying the observed effects.

When the gut barrier function is compromised, the resulting increased local antigen exposure could trigger the host’s immune system, thus leading to the onset of an inflammatory state [80, 81]. As expected, the functional alterations found were compassed with altered inflammatory status in very early life. Furthermore, CSD pups have a decrease in colonic IL-10 cytokine, an important immunoregulatory cytokine that suppresses exacerbated mucosal immune response due to colonic inflammation and preserves the intestinal mucus barrier [82, 83]. These differences, that were restored at weaning, confirm the presence of a transitory inflammatory status in very early life. Besides, an increased abundance in Th1, Th2 and Th17 cells was observed in spleen at weaning, with Th-1 cells also found to increase in MLN, confirming the local pro-inflammatory Th-1 profile of the CSD pups in the early life. Although the decrease in IL-10 highlights the possible deficit in regulatory T cells, and others have found a downregulation of regulatory markers in CSD mice [49], we did not observe significant changes in the Treg levels.

GF mice were colonized by faecal microbiota transplantation using VD and CSD mice samples collected at weaning to address the causality of the microbiota on the phenotypes observed. Mice were colonized after weaning to directly address the phenotype unchained by the microbiota administered and do not interact with the weaning reaction process [22]. GF-CSD mice were more susceptible to induced intestinal inflammation than GF-VD ones, confirming the causal role of the gut microbiota. However, as expected, not all the phenotypes were transferred with the microbiota, probably because some early life priming process was already successfully performed without microbiota in these mice. GF-CSD mice showed transient alterations in gut structure and permeability as it happens in CSD mice, with an increase in AB^+^ GC and a decrease of PAS^+^ GC confirming that the alteration of GC populations is microbiota driven. Finally, GF-CSD showed the same decreased butyrate/acetate ratio than CSD mice.

In the last years, several studies suggested the use of interventional methods to restore CSD-induced dysbiosis [13, 84]. In our study, supplementation of GF-CSD mice with lactobacilli prevented the increased susceptibility to chemically induced inflammation observed in GF-CSD mice. GF-CSD + Lb mice showed a restoration of most of the parameters altered by CSD, except for SCFA production, as GF-CSD + Lb mice counterbalanced butyrate deficit by increasing propionate and isobutyrate concentrations. Isobutyrate could function as a carbon source for energy in colonocytes under conditions of defective butyrate oxidation or low butyrate availability, as it happens in GF-CSD mice [70]. On its side, propionate has been found to exert anti-inflammatory properties, improving intestinal barrier function and reducing pro-inflammatory factors TNF-α, IL-1β and IL-6 mRNA in colon [71]. All these facts point to a compensatory mechanism at the SCFA level in which reduced butyrate is counterbalanced by propionate and isobutyrate increases which partially replace butyrate functions.

## Conclusions

Our results suggest that excessive exposure to a very diverse microbiota too early in life has detrimental consequences on host homeostasis in the short and long term in mice. This “too much too early” principle due to the presence of increased microbial diversity in the very early life involves excessive exposure to bacterial antigen across the vulnerable gut barrier in the first days of life, before the gut closure. These premature microbial stimuli affect the gut barrier by altering epithelial structure and mucus production, disrupting gut homeostasis by increasing local antigen exposure, possibly related to an altered acetate/butyrate ratio, which could trigger the host’s immune system, thus determining the onset of an inflammatory state. It also switches pups toward a pro-inflammatory Th1 immune response. Further experiments should be performed to evaluate other possible impacts of the CDS-induced alteration of the gut barrier dysfunction in other parameters, such as mucosal IgA or AMP production. Furthermore, in mice, this mechanism could be the linchpin behind the phenotypic effects that lead to major susceptibility to induced inflammation later in life, as it has been confirmed in our study with chemically induced colitis models. Besides, microbiota has been found to be causal in most of the phenotypical parameters found, as shown by experiments with ex GF mice. Finally, the microbiota modulation strategy tested here has shown positive effects in our murine model. It is important to remark that the transferability of our results to the human context should be confirmed as our murine model, as all the models, present dissimilarities with the modelled system [50].

### Supplementary Information

**Additional file 1: Fig. S1.** Microbiota analysis. Total sample profile at (A) phylum and (B) genus level. (C) Non-Metric Multidimensional Scaling (NMDS) analyses based on Bray-Curtis dissimilarities of the whole dataset. (D) Heat-map of OTU counts by time point. (E) Alpha and beta diversity (measured by Chao1 and Shannon index) and richness by time point.

**Additional file 2: Fig. S2.** Microbiota compositional differences. Phylum-level differences between CSD and VD at 5 days (A), 6 weeks (B), second injection (C) and endpoint (D). Groups: vaginal delivery (VD, blue) and C-section delivery (CSD, red).

**Additional file 3: Fig. S3.** Metagenomic functional prediction by PICRUSt. Heatmap and PCoA representation of the metagenomic functions inferred from the phylogenetic profiles using PCRUSt at 5 days (A), weaning (B) and 6 weeks (C). Groups: vaginal delivery (VD, blue) and C-section delivery (CSD, red).

**Additional file 4: Fig. S4.** Metagenomic functional prediction during DNBS-induced chronic colitis. Heatmap and PCoA representation of metagenomic functions inferred from the phylogenetic profiles by PCRUSt before second injection of DNBS (A, B) and endpoint (C, D) in vehicle (A, C) and inflamed (B, D) groups. Groups: vaginal delivered mice, non-inflamed (VD-vehicle, light blue), vaginal delivered mice, inflamed (VD-DNBS, dark blue), C-section delivered mice, non-inflamed (CSD-vehicle, light red), C-section delivered mice, inflamed (CSD-DNBS, dark red).

**Additional file 5: Table S1.** OTUs modulated by CSD at 5 days.

**Additional file 6: Table S2.** OTUs modulated by CSD at weaning.

**Additional file 7: Table S3.** OTUs modulated by CSD at 6 weeks.

**Additional file 8: Table S4.** Bacterial genera modulated by CSD at 5 days.

**Additional file 9: Table S5.** Butyrate producers relative abundances.

**Additional file 10: Table S6.** Genes modulated by CSD at weaning.

## Data Availability

The 16S RNA raw reads are available at NCBI’s SRA repository (BioProject PRJNA876103; accessions SAMN30638467 to SAMN30638852). Microarray data are deposited in GEO under the accession number GSE214311.

## Abbreviations

AB: Alcian blue
AbfD: Vinylacetyl-CoA 3,2-isomerase
ATO: Butyryl-CoA:acetate CoA transferase
AUC: Area under the curve
BUK: Butyrate kinase
CD: Crohn’s disease
CFU: Colony-forming units
CSD: C-section delivery
CSS: Cumulative sum scaling
DMEM: Dulbecco’s Modified Eagle’s Medium
DNBS: 2,4-Dinitrobenzene sulphonic acid
FBS: Foetal bovine serum
FDR: False discovery rate
FSA: Fluorescein sulphonic acid
GC: Goblet cells
GF: Germ-free
GM-CSF: Granulocyte-macrophage colony-stimulating factor
HES: Hematoxylin-eosin-saffron
HRP: Horseradish peroxidase
IBD: Inflammatory bowel diseases
KO: KEGG Orthology
Lcn-2: Lipocalin-2
MCP-1: Monocyte chemoattractant protein-1
MPO: Myeloperoxidase activity
MRS: Man, Rogosa and Sharpe media
NMDS: Nonmetric multidimensional scaling
OTU: Operational taxonomic unit
PAS: Periodic acid-Schiff
PCoA: Principal coordinates analysis
SCFAs: Short-chain fatty acids
SPCIT: Sparse partial correlation and information theory
SPF: Specific pathogen-free
TDA: TRITC-dextran
VD: Vaginal delivery
