# Correction: Gut barrier-microbiota imbalances in early life lead to higher sensitivity to inflammation in a murine model of C-section delivery

**DOI:** 10.1186/s40168-023-01631-w

**Published:** 2023-08-05

**Authors:** M. Barone, Y. Ramayo‑Caldas, J. Estellé, K. Tambosco, S. Chadi, F. Maillard, M. Gallopin, J. Planchais, F. Chain, C. Kropp, D. Rios‑Covian, H. Sokol, P. Brigidi, P. Langella, R. Martín

**Affiliations:** 1 Microbiomics Unit, Department of Medical and Surgical Sciences, University of Bologna, 40138 Bologna, Italy; 2 INRAE, AgroParisTech, GABI, Paris-Saclay University, 78350 Jouy-en-Josas, France; 3 Animal Breeding and Genetics Program, Institute for Research and Technology in Food and Agriculture (IRTA), Torre Marimon, 08140 Caldes de Montbui, Spain; 4 INRAE, AgroParisTech, Micalis Institut, Paris-Saclay University, 78350 Jouy-en-Josas, France; 5 CNRS, CEA, l’Institut de Biologie Intégrative de La Cellule (I2BC), Paris-Saclay University, 91405 Orsay, France; 6 Gastroenterology Department, Centre de Recherche Saint-Antoine, Centre de Recherche Saint-Antoine, CRSA, AP-HP, INSERM, Saint Antoine Hospital, Sorbonne Université, 75012 Paris, France; 7 Paris Centre for Microbiome Medicine (PaCeMM) FHU, Paris, France

**Correction: Microbiome 11, 140 (2023)**

**https://doi.org/10.1186/s40168-023-01584-0**

Following the publication of the original article [1], the author reported that in Fig. 5, the histology photos are only white boxes, there are no images. The correct Fig. 5 has been included here and the original article has been updated.

**Fig. 5:**
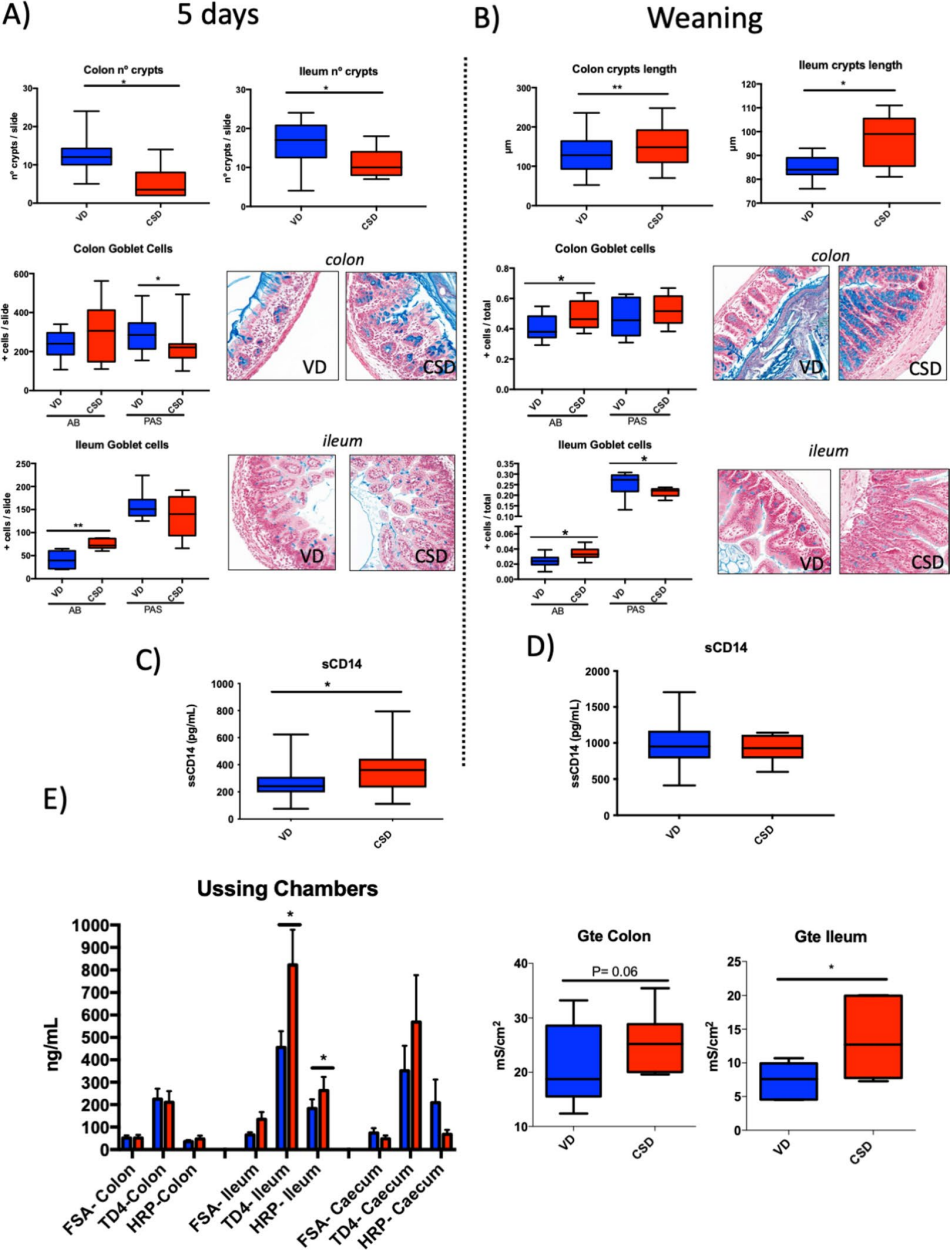
Gut barrier structure and permeability features at 5 days and weaning. **A** Colon or ileum number of crypts at 5 days (*n* = 7–8); percentage of goblet cells along with representative photos of colon and ileum samples, stained by Alcian blue or PAS. **B** Crypt length and goblet cell percentages along with representative photos of colon or ileum samples, stained by AB or PAS at weaning (*n* = 6–10). **C** and **D** Concentration of sCD14 in serum samples at 5 days (*n* = 20) and weaning (*n* = 20). **E** Global permeability measured by the tracer FITC-dextran in serum at weaning (*n* = 28). Permeability to the tracers FSA, TD4 and HRP of colon, ileum and caecum tissues mounted in Ussing chambers (*n* = 10). Electrical conductance of colon and ileum tissues mounted in Ussing chambers (*n* = 10). Groups: vaginal delivery (VD, blue) and C-section delivery (CSD, red). AB, Alcian blue; PAS, periodic acid-Schiff. *p-value < 0.05; **p-value < 0.01
