# Supplementary figures and images for: Gut barrier-microbiota imbalances in early life lead to higher sensitivity to inflammation in a murine model of C-section delivery
Source: Microbiome. 2023 Jul 3;11:140. doi: 10.1186/s40168-023-01584-0 (PMC10316582; doi:10.1186/s40168-023-01584-0)

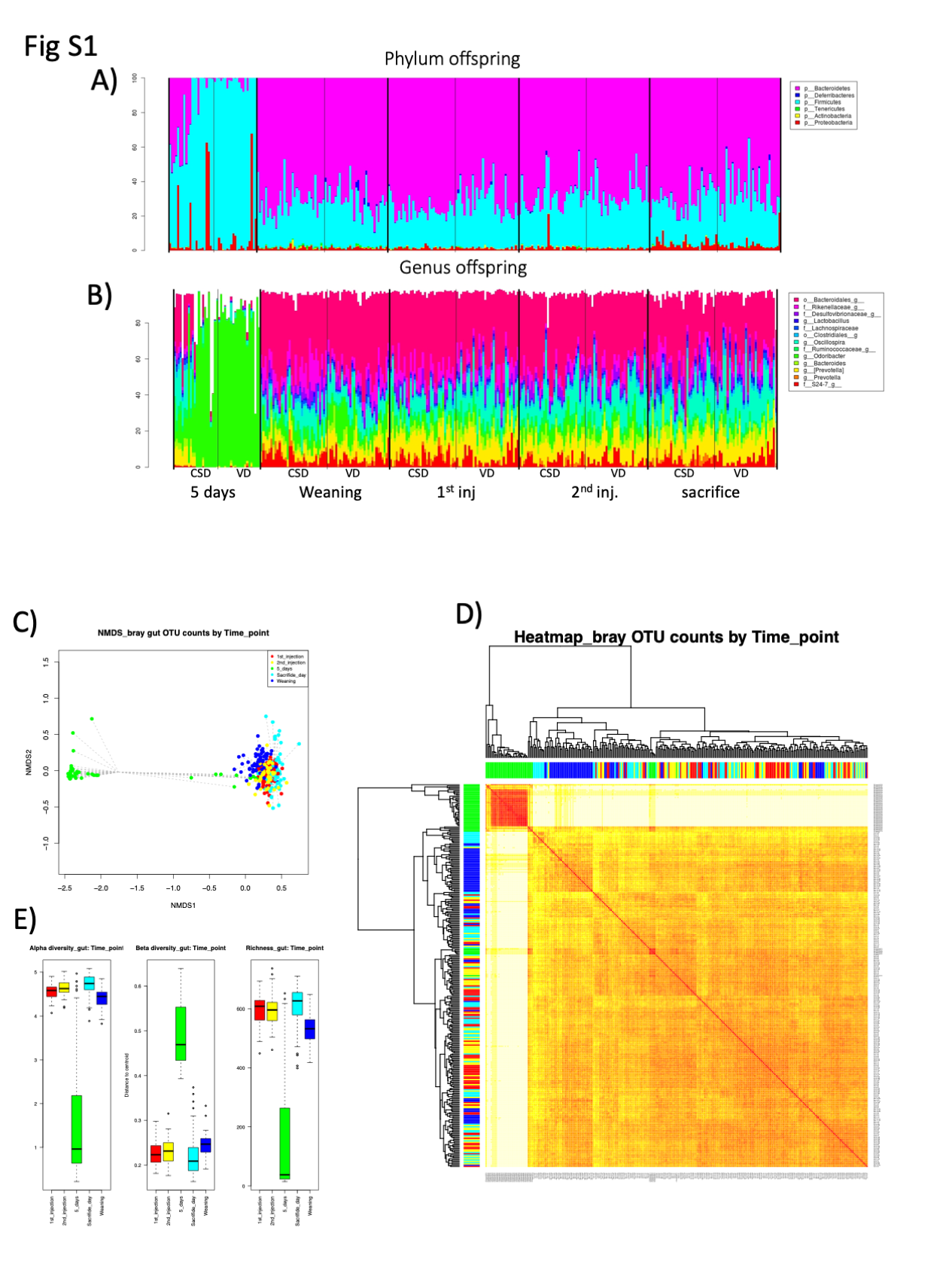

Supplement: Supplementary file 2 — Additional file 1: Fig. S1. Microbiota analysis. Total sample profile at (A) phylum and (B) genus level. (C) Non-Metric Multidimensional Scaling (NMDS) analyses based on Bray-Curtis dissimilarities of the whole dataset. (D) Heat-map of OTU counts by time point. (E) Alpha and beta diversity (measured by Chao1 and Shannon index) and richness by time point. [file 40168_2023_1584_MOESM1_ESM.tiff]

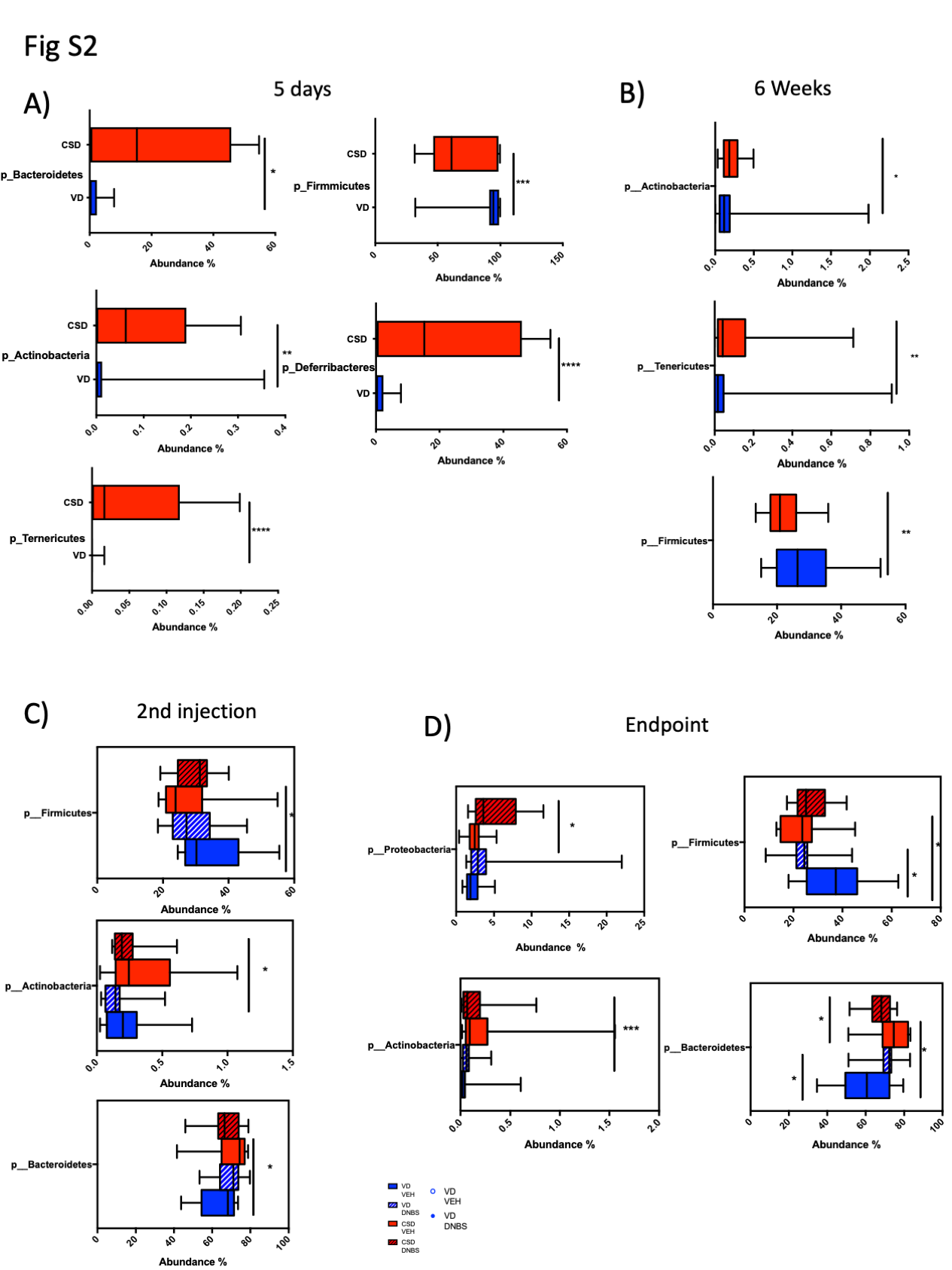

Supplement: Supplementary file 3 — Additional file 2: Fig. S2. Microbiota compositional differences. Phylum-level differences between CSD and VD at 5 days (A), 6 weeks (B), second injection (C) and endpoint (D). Groups: vaginal delivery (VD, blue) and C-section delivery (CSD, red). [file 40168_2023_1584_MOESM2_ESM.tiff]

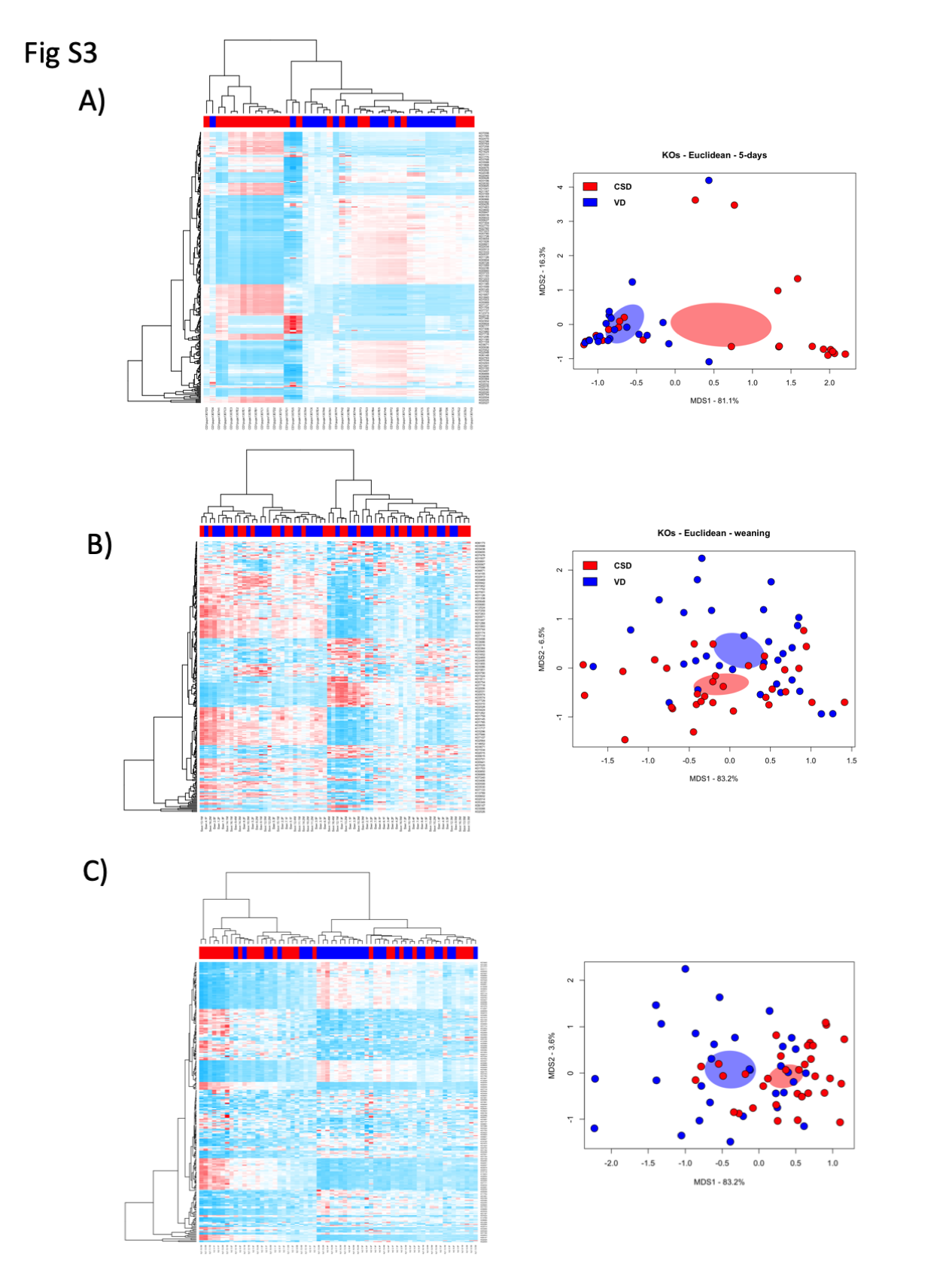

Supplement: Supplementary file 4 — Additional file 3: Fig. S3. Metagenomic functional prediction by PICRUSt. Heatmap and PCoA representation of the metagenomic functions inferred from the phylogenetic profiles using PCRUSt at 5 days (A), weaning (B) and 6 weeks (C). Groups: vaginal delivery (VD, blue) and C-section delivery (CSD, red). [file 40168_2023_1584_MOESM3_ESM.tiff]

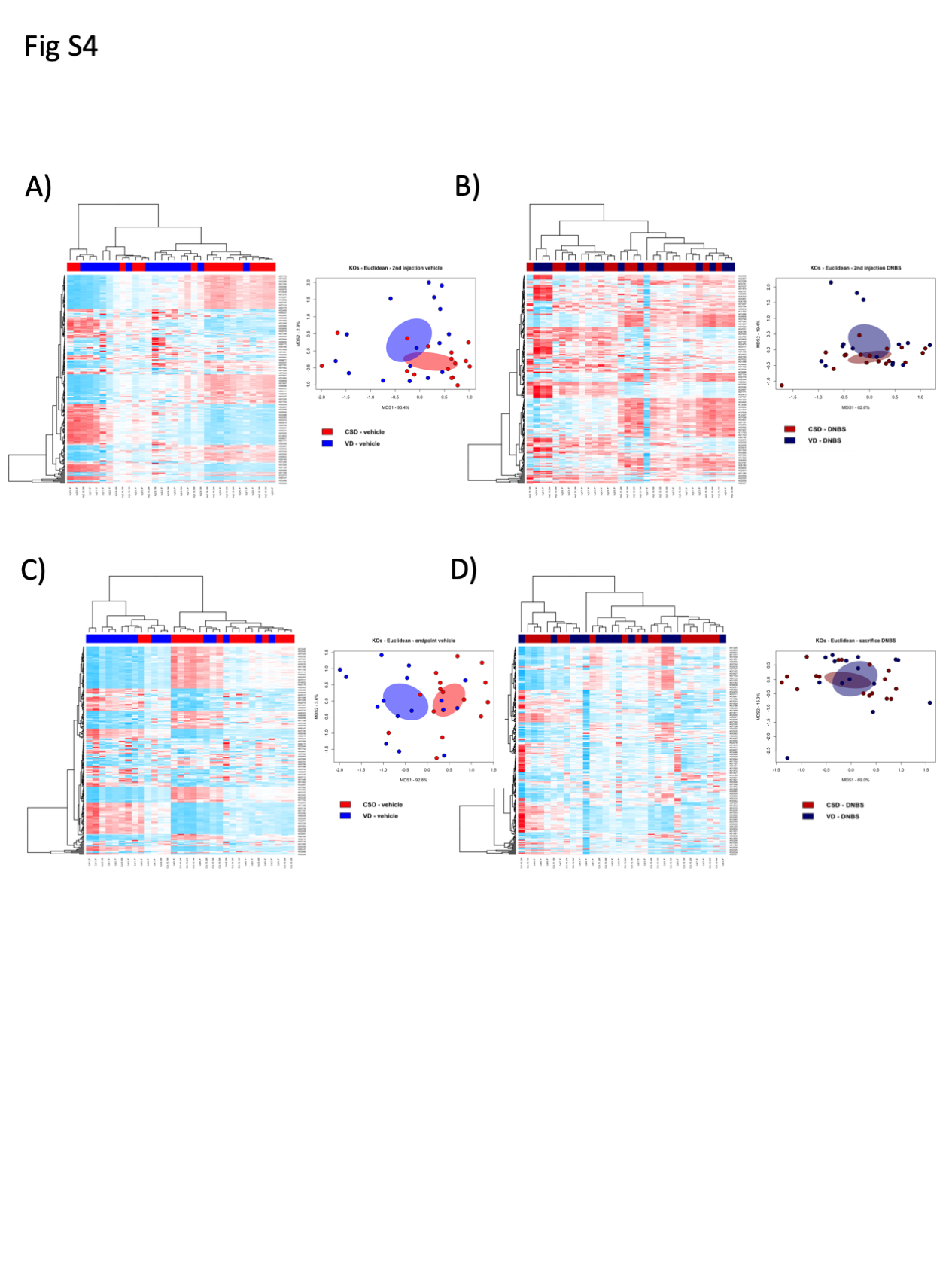

Supplement: Supplementary file 5 — Additional file 4: Fig. S4. Metagenomic functional prediction during DNBS-induced chronic colitis. Heatmap and PCoA representation of metagenomic functions inferred from the phylogenetic profiles by PCRUSt before second injection of DNBS (A, B) and endpoint (C, D) in vehicle (A, C) and inflamed (B, D) groups. Groups: vaginal delivered mice, non-inflamed (VD-vehicle, light blue), vaginal delivered mice, inflamed (VD-DNBS, dark blue), C-section delivered mice, non-inflamed (CSD-vehicle, light red), C-section delivered mice, inflamed (CSD-DNBS, dark red). [file 40168_2023_1584_MOESM4_ESM.tiff]
