# Supplementary material for: Gut barrier-microbiota imbalances in early life lead to higher sensitivity to inflammation in a murine model of C-section delivery
Source: Microbiome. 2023 Jul 3;11:140. doi: 10.1186/s40168-023-01584-0 (PMC10316582; doi:10.1186/s40168-023-01584-0)
Supplement: Supplementary file 10 — Additional file 9: Table S5. Butyrate producers relative abundances. [file 40168_2023_1584_MOESM9_ESM.docx]

| **Genus** | **Day 5** | **Weaning** | **6 weeks** |
| --- | --- | --- | --- |
| *Odoribacter* | -2.46 (1.65) ^NS^ | 1.63 (0.34) *** | 0.43 (0.24) ^NS^ |
| *Coprococcus* | -4.48 (1.19) *** | 0.19 (0.27) ^NS^ | 0.77 (0.35) * |
| *Roseburia* | -2.75 (0.78) ** | 0.70 (0.44) ^NS^ | 1.7 (0.77) * |
| *Anaerostipes* | -3.31 (0.75) ** | -0.54 (0.87) ^NS^ | 0.80 (0.98) ^NS^ |
| *Clostridium* | -1.15 (0.68) ^NS^ | -0.33 (0.71) ^NS^ | -0.22 (0.74) ^NS^ |
